# Systemic GDF11 attenuates depression-like phenotype in aged mice via stimulation of neuronal autophagy

**DOI:** 10.1038/s43587-022-00352-3

**Published:** 2023-02-02

**Authors:** Carine Moigneu, Soumia Abdellaoui, Mariana Ramos-Brossier, Bianca Pfaffenseller, Bianca Wollenhaupt-Aguiar, Taiane de Azevedo Cardoso, Claire Camus, Aurélie Chiche, Nicolas Kuperwasser, Ricardo Azevedo da Silva, Fernanda Pedrotti Moreira, Han Li, Franck Oury, Flávio Kapczinski, Pierre-Marie Lledo, Lida Katsimpardi

**Affiliations:** 1 Perception and Memory Lab, Institut Pasteur, Université Paris Cité, CNRS UMR3571, Paris, France; 2 Institut Necker Enfants Malades, INSERM UMR-S1151, Université Paris Cité, Paris, France; 3 Department of Psychiatry and Behavioural Neurosciences, McMaster University, Hamilton, ON Canada; 4 Cellular Plasticity in Age-Related Pathologies Laboratory, Institut Pasteur, Université Paris Cité, CNRS UMR3738, Paris, France; 5 Department of Health and Behavior, Catholic University of Pelotas, Pelotas, Brazil; 6 Instituto Nacional de Ciência e Tecnologia Translacional em Medicina (INCT-TM), Porto Alegre, Brazil; 7 Department of Psychiatry, Universidade Federal do Rio Grande do Sul (UFRGS), Porto Alegre, Brazil; 8 Present Address: Institut Necker Enfants Malades, INSERM UMR-S1151, Université Paris Cité, Paris, France

**Keywords:** Neuroscience, Cognitive ageing

## Abstract

Cognitive decline and mood disorders increase in frequency with age. Many efforts are focused on the identification of molecules and pathways to treat these conditions. Here, we demonstrate that systemic administration of growth differentiation factor 11 (GDF11) in aged mice improves memory and alleviates senescence and depression-like symptoms in a neurogenesis-independent manner. Mechanistically, GDF11 acts directly on hippocampal neurons to enhance neuronal activity via stimulation of autophagy. Transcriptomic and biochemical analyses of these neurons reveal that GDF11 reduces the activity of mammalian target of rapamycin (mTOR), a master regulator of autophagy. Using a murine model of corticosterone-induced depression-like phenotype, we also show that GDF11 attenuates the depressive-like behavior of young mice. Analysis of sera from young adults with major depressive disorder (MDD) reveals reduced GDF11 levels. These findings identify mechanistic pathways related to GDF11 action in the brain and uncover an unknown role for GDF11 as an antidepressant candidate and biomarker.

## Abstract (web summary)

Moigneu, Abdellaoui and colleagues show that GDF11 attenuates depression-like behavior and improves memory in aged mice through neuronal autophagy and mTOR. Serum levels of GDF11 are inversely associated with depression in patients.

## Main

Aging is often accompanied by severe cognitive impairments, memory loss and age-related depression^1^. Independently of age, MDD affects around 20% of the population and correlates with short-term and working memory deficits, which exacerbate the effects of aging in some older adults^2^. Depression can resemble a state of accelerated aging as depressed individuals often exhibit a higher incidence of age-associated diseases, including Alzheimer’s and other neurodegenerative diseases^3^.

The hippocampus plays a key role in the regulation of memory and depression. Small hippocampal volumes in humans have been associated with MDD, other psychiatric disorders and memory impairments, establishing the hippocampus as a structural link among aging, mood disorders and memory decline^4^. In animal models, depression-like behavior is also associated with structural and functional alterations of the hippocampus and is most often related to impaired neurogenesis^5^. At the cellular level, hippocampal neurogenesis is crucial for memory formation and cognitive function, whereas impaired adult neurogenesis has been linked to memory decline and depression-like phenotypes^6,7^.

Infusion of youthful blood factors or blocking proaging factors has been shown to attenuate age-related impairments in neurogenesis, memory decline and olfactory perception^8–13^. One of these blood factors is growth differentiation factor 11 (GDF11), a member of the transforming growth factor beta (TGF-β) superfamily, which has a critical role in embryonic development as a key regulator of patterning and formation of several tissues^14–19^. The role of GDF11 in the developing central nervous system regulates the progression of neurogenesis as well as differentiation of neural subtypes^19–22^. In adults, GDF11 was recently shown to negatively regulate neurogenesis^23^. Because circulating levels of GDF11 decline with age^24,25^, supplementation with recombinant GDF11 (rGDF11) in aged mice was previously shown to rejuvenate neurogenesis in both the subventricular zone and hippocampal dentate gyrus (DG) and improve cerebral vasculature^8,26^. Despite increasing reports on the various effects of GDF11, it remains unknown whether GDF11 improves cognitive impairments, as well as its precise mechanism of action in the brain.

Here, we report that systemic GDF11 treatment in aged mice was sufficient to prevent memory decline and depression-like behavior, enhance hippocampal neurogenesis and autophagy and reduce hippocampal senescence. Mechanistically, GDF11 stimulated neuronal autophagy and inhibited the mTOR pathway in a neurogenesis-independent manner. Using the corticosterone (CORT)-induced murine model of depression-like behavior, we demonstrated that GDF11 inhibited a depression-like phenotype in young mice. Finally, we revealed that GDF11 levels were decreased in young adults with MDD or presenting a current depressive episode, making it a potential biomarker of depression and a possible agent for therapeutic interventions.

## Results

### Systemic GDF11 administration restores memory decline and attenuates the depression-like phenotype

**Fig. 1:**
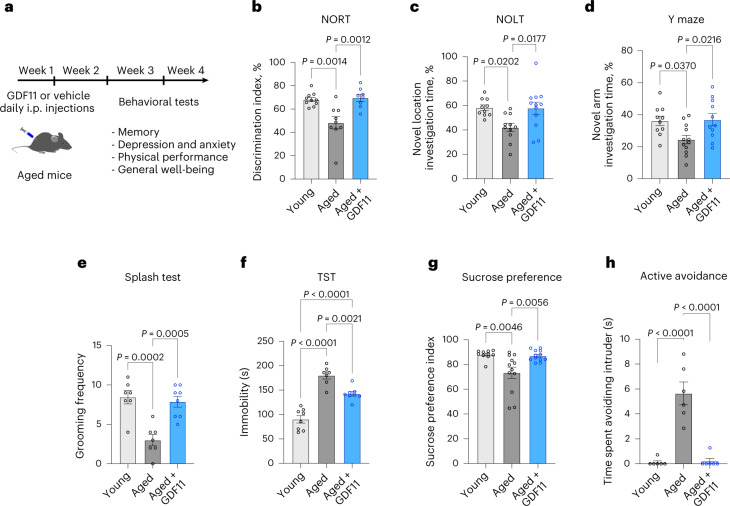
Systemic GDF11 administration restores memory decline and depression-like phenotype in aged mice. **a**, Schematic representation of the experimental procedure. **b**, Measurement of discrimination index during the NORT (percentage of time spent to observe the novel object divided by the total investigation time for both objects) (*n*_Young_ = 10 mice, *n*_Aged_ = 9 mice, *n*_Aged+GDF11_ = 8 mice; F (2, 24) = 10.9). **c**, Measurement of percent investigation time of the novel location during the novel object location test (NOLT) (percentage of time spent to observe the novel location of the object divided by the total investigation time for both objects) (*n*_Young_ = 10 mice, *n*_Aged_ = 11 mice, *n*_Aged+GDF11_ = 12 mice; F (2, 30) = 5.5). **d**, Measurement of percent investigation time of the novel arm during the Y-maze test (percentage of time spent to observe the novel arm divided by the total investigation time for both arms) (*n*_Young_ = 10 mice, *n*_Aged_ = 12 mice, *n*_Aged+GDF11_ = 11 mice; F (2, 30) = 5). **e**, Measurement of grooming frequency during the Splash test (*n*_Young_ = 7 mice, *n*_Aged_ = 7 mice, *n*_Aged+GDF11_ = 8 mice; F (2, 19) = 15.3). **f**, Measurement of immobility time during the TST (*n*_Young_ = 8 mice, *n*_Aged_ = 8 mice, *n*_Aged+GDF11_ = 7 mice; F (2, 20) = 45.5). **g**, Measurement of the sucrose preference index over two days (volume consumed from the sucrose bottle divided by the total volume consumed) (*n*_Young_ = 10 mice, *n*_Aged_ = 12 mice, *n*_Aged+GDF11_ = 12 mice; F (2, 31) = 7.8). **h**, Measurement of the time spent avoiding the intruder during the social interaction test (*n*_Young_ = 6 mice, *n*_Aged_ = 6 mice, *n*_Aged+GDF11_ = 6 mice; F (2, 15) = 33.7). One-way analysis of variance (ANOVA) and Tukey’s post hoc test for multiple comparisons; F (DFn, DFd) values presented for each ANOVA statistical analysis; *P* values <0.05 are represented on the graph; mean ± standard error of the mean (s.e.m.). Source data

We treated 22-month-old C57BL/6JRj male mice with rGDF11 via daily intraperitoneal (i.p.) injections at a dose of 1 mg kg^−1^, as previously described^24^. Aged-matched controls and young (3-month-old) mice were injected with saline. Mice were injected over the course of 3 weeks, and behavioral tests were performed on the third week (Fig. 1a). Assessing memory using the novel object recognition test (NORT) showed that aged mice were unable to discriminate the novel object and scored significantly lower than young mice (aged, 48.2% ± 5.5; young, 68.2 ± 1.7; Fig. 1b). On the contrary, GDF11-treated aged mice presented a significant 44.2% increase in the novel object discrimination index (GDF11 = 69.6 ± 3%; aged, 48.2% ± 5.5; Fig. 1b). Hippocampus-dependent spatial memory was specifically assessed with the novel object location test, where aged mice spent significantly less time investigating the novel location compared to both young and aged GDF11-treated mice (aged, 42 ± 3.3%; young = 58.2 ± 2.2%; GDF11 = 57.8 ± 5%; Fig. 1c). In the Y-maze test (used to assess recognition memory), young and GDF11-treated aged mice spent significantly more time investigating the novel arm of the maze than aged mice (aged, 24.4 ± 2.7%; young, 36.1 ± 2.9%; GDF11 = 36.9 ± 3.7%), confirming the effect of GDF11 in improving age-related memory impairments (Fig. 1d).

Subsequently, young and aged mice were subjected to a series of behavioral tests aimed at assessing the level of a depression-like phenotype, often associated with advanced aging. During the splash test, aged mice exhibited a significant 2.8-fold decrease in grooming frequency compared to young mice (aged, 3 ± 0.7; young, 8.4 ± 0.8), confirming the known effect of aging on depression^27^ (Fig. 1e). On the contrary, aged GDF11-treated mice showed a significant 2.5-fold increase in grooming frequency compared to aged mice (Fig. 1e). The tail suspension test (TST), which measures despair-like behavior, showed that aged mice remained immobile twice as long as young mice (aged, 180 ± 7.3 s; young, 90 ± 7.6 s) and 1.5-times longer than aged GDF11-treated mice (aged, 180 ± 7.3 s; GDF11,142 ± 4.7 s; Fig. 1f). Overall, the score of GDF11-treated mice suggests improved symptoms of depression-like phenotype. Next, we tested anhedonia, another trait of depression-like phenotype, with the sucrose preference test and found that aged mice showed significantly decreased preference for the sucrose solution compared to young (aged, 73.2 ± 4.5; young, 87.9 ± 1.2), but GDF11 treatment in aged mice restored this preference (GDF11, 86.9 ± 1.2; aged, 73.2 ± 4.5; Fig. 1g). Finally, the measurement of intruder avoidance during the social interaction test revealed that both young and aged GDF11-treated mice spent less time avoiding the intruder than aged mice (aged, 5.6 ± 0.9 s; GDF11, 0.2 ± 0.2 s; young, 0.1 ± 0.1 s; Fig. 1h).

**Extended Data Fig. 1:**
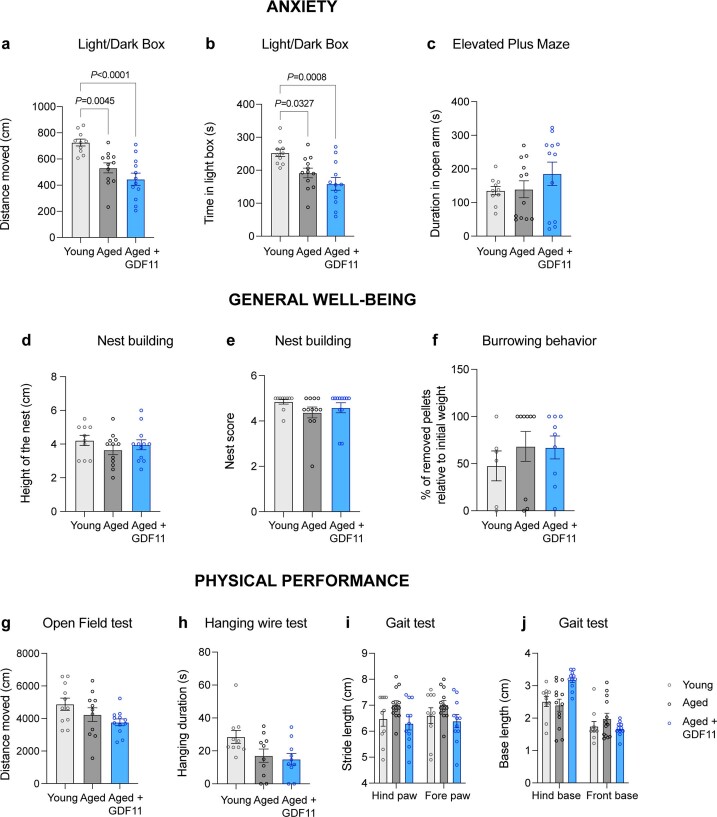
GDF11 treatment does not affect anxiety-like state, general well-being or physical performance. (**a**–**c**) Behavioral tests related to anxiety: (**a**) Measurement of the distance moved in the Light Box, during the Light/Dark Box test (n_Young_ = 10 mice, n_Aged_ = 10 mice, n_Aged+GDF11_ = 12 mice; F (2, 31) = 12.8), (**b**) Measurement of the time spent in the Light Box, during the Light/Dark Box test (n_Young_ = 10 mice, n_Aged_ = 10 mice, n_Aged+GDF11_ = 12 mice; F (2, 31) = 8.5), (**c**) Measurement of the time spent in the open arms, during the Elevated Plus Maze test (n_Young_ = 10 mice, n_Aged_ = 10 mice, n_Aged+GDF11_ = 12 mice; F (2, 31) = 1.0). (**d**–**f**) Behavioral tests related to general well-being: (**d**) Measurement of the height of the nest (n_Young_ = 10 mice, n_Aged_ = 10 mice, n_Aged+GDF11_ = 12 mice; F (2, 31) = 0.8), (e) Scoring of the nest quality based on five criteria (n_Young_ = 10 mice, n_Aged_ = 10 mice, n_Aged+GDF11_ = 12 mice; F (2, 31) = 1.2), (**f**) measurement of the burrowing index (percentage of weight of pellets removed from the burrow divided by total pellet weight), during the Burrowing test (n_Young_ = 6 mice, n_Aged_ = 9 mice, n_Aged+GDF11_ = 9 mice; F (2, 21) = 0.5). (**g**–**j**) Behavioral tests related to physical performance: (**g**) measurement of the distance moved during the Open Field test (n_Young_ = 11 mice, n_Aged_ = 11 mice, n_Aged+GDF11_ = 12 mice; F (2, 31) = 2.7), (**h**) measurement of the time mice spent hanging on the wire during the Hanging Wire test (n_Young_ = 10 mice, n_Aged_ = 10 mice, n_Aged+GDF11_ = 10 mice; F (2, 27) = 3.6), (**i**) measurement of the hind paw and fore paw stride length during the Gait test (n_Young_ = 10 mice, n_Aged_ = 13 mice, n_Aged+GDF11_ = 11 mice; F (2, 62) = 0.03), (**j**) measurement of the hind base and front base length during the Gait test (n_Young_ = 10 mice, n_Aged_ = 13 mice, n_Aged+GDF11_ = 11 mice; F (2, 62) = 7.4). One-way and two-way ANOVA and Tukey’s post hoc test for multiple comparisons; F (DFn, DFd) value presented for each ANOVA statistical analysis; *P* values < 0.05 are represented on the graph; mean ± s.e.m. Source data

Given the above results, we next asked whether GDF11 would also influence anxiety-like state. Using the light/dark box (LDB) test, we found that young mice covered a significantly longer distance (young, 726 ± 27 cm; aged, 532 ± 38 cm) and spent more time (young, 252 ± 11.4 s; aged, 191.9 ± 14.5 s) in the light box than aged mice (Extended Data Fig. 1a,b). However, GDF11 treatment had no effect compared to control in aged-matched mice (distance: GDF11, 445.7 ± 46.2 cm; aged, 531.9 ± 37.9 cm; time: GDF11,158.6 ± 19.3 s; aged, 191.9 ± 14.5 s; Extended Data Fig. 1a,b). Next, the elevated plus maze (EPM) test, for anxiety-like phenotype, revealed no difference between aged control and aged GDF11-treated mice in the duration/time spent in open arms (GDF11, 185.5 ± 34.6 s; aged, 139.4 ± 25 s), despite the trend of a subgroup of GDF11-treated mice exceeding the mean duration (Extended Data Fig. 1c). Collectively, these results show that GDF11 has a distinct effect on the depression-like phenotype.

To examine whether GDF11 has a broader impact on behavior, we performed additional assays targeting general well-being and physical performance. First, we measured nest building as a general index of well-being by assessing nest height and building scores. Overall, the mice showed the same capacities for nest building (Extended Data Fig. 1d,e). Next, we evaluated burrowing behavior and found that all mice behaved similarly (Extended Data Fig. 1f). Using the open field test to assess physical performance, we measured the total distance traveled and found no difference between GDF11 or vehicle treatment in aged mice (Extended Data Fig. 1g). We assessed strength and endurance with the hanging wire test and saw no difference regardless of age or treatment (Extended Data Fig. 1h). Then, we performed the gait test to evaluate possible locomotion problems. Once again, we found no significant difference between the groups. These results demonstrate that GDF11 in aged mice has no effect on physical performance. Therefore, the vast panel of behavioral tests demonstrates that GDF11 administration specifically improves memory and depression-like phenotype.

### Cellular changes induced by GDF11 treatment

We next sought to identify the cellular mechanisms underlying GDF11 treatment by analyzing the brains of young and aged mice at 9 days of treatment (the earliest time point where we previously saw increased olfactory neurogenesis), for hallmarks of aging, including neurogenesis decline, increased senescence and autophagy impairments.

**Fig. 2:**
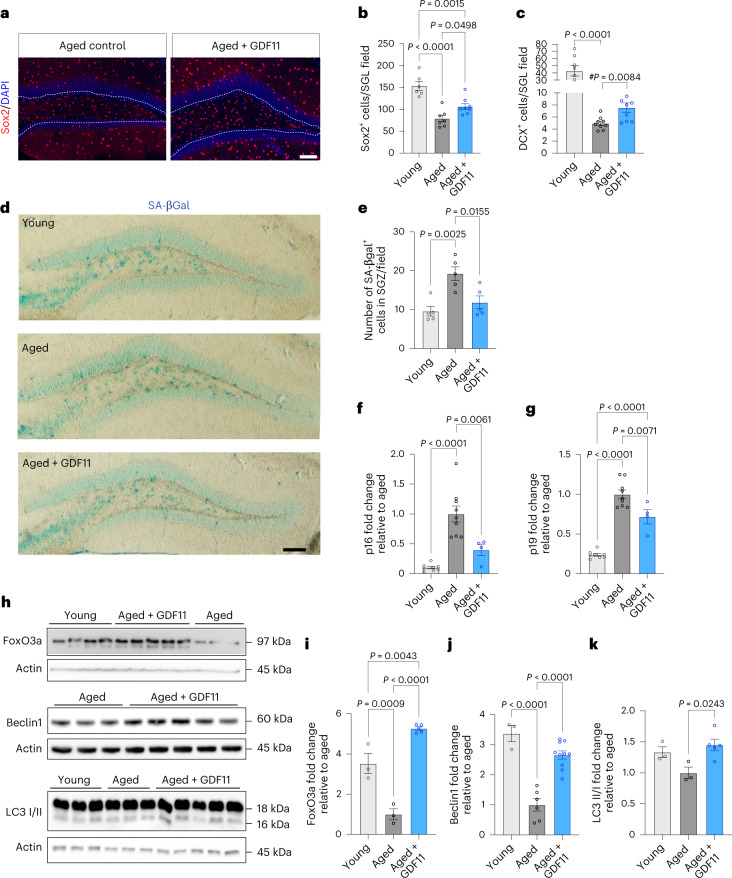
Cellular changes induced by GDF11 treatment in the brains of aged mice. **a**, Representative confocal images of the DG of the hippocampus of young, aged and aged-GDF11 mice immunostained for Sox2 (red) and DAPI (blue). Scale bar: 100 μm. **b**, Quantification of Sox2^+^ NSCs in the SGL of the DG (*n*_Young_ = 6 mice, *n*_Aged_ = 7 mice, *n*_Aged+GDF11_ = 8 mice; F (2, 18) = 21). **c**, Quantification of DCX^+^ neuroblasts in the SGL of the DG (*n*_Young_ = 8 mice, *n*_Aged_ = 9 mice, *n*_Aged+GDF11_ = 9 mice; F (2, 23) = 30.3; #*P* value by Mann–Whitney test between aged and aged + GDF11). **d**, Representative images of SA-βGal staining in the DG of young, aged and aged-GDF11 mice. Scale bar: 100 μm. **e**, Quantification of SA-βGal^+^ cells in the SGZ of the DG (n = 5 mice per group; F (2, 12) = 10.3). **f**,**g**, Real-time qPCR for hallmarks of senescence showing fold changes in mRNA levels of p16 (f) (*n*_Young_ = 8 mice, *n*_Aged_ = 9 mice, *n*_Aged+GDF11_ = 4 mice; F (2, 18) = 22.2) and p19 (g) (*n*_Young_ = 8 mice, *n*_Aged_ = 9 mice, *n*_Aged+GDF11_ = 4 mice; F (2, 18) = 68) in hippocampi of young, aged and aged-GDF11 mice, relative to aged mice. **h**, Representative Western blot images of hippocampal lysates from young, aged and GDF11-treated aged mice after 9 days of treatment. **i**–**k**, Quantification of western blots by optical intensity for Foxo3a (i) (*n*_Young_ = 3 mice, *n*_Aged_ = 3 mice, *n*_Aged+GDF11_ = 5 mice; F (2, 8) = 63), Beclin 1 (j) (*n*_Young_ = 3 mice, n_Aged_ = 6 mice, *n*_Aged+GDF11_ = 10 mice; F (2, 16) = 34.9), and LC3 (k) (*n*_Young_ = 3 mice, *n*_Aged_ = 3 mice, *n*_Aged+GDF11_ = 5 mice; F (2, 8) = 5.7). One-way ANOVA and Tukey’s post hoc test for multiple comparisons; F (DFn, DFd) values presented for each ANOVA statistical analysis; *P* values < 0.05 are represented on the graph; mean ± s.e.m. Source data

**Extended Data Fig. 2:**
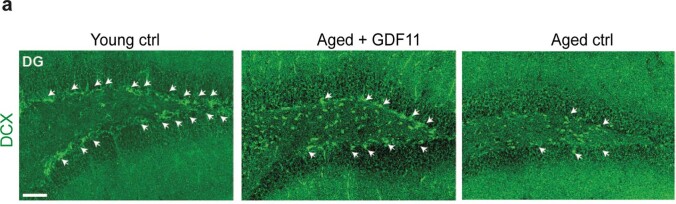
DCX expression in the DG after GDF11 treatment. (**a**) Representative confocal images of the dentate gyrus of the hippocampus immunostained for DCX (doublecortin, a marker of immature neuroblasts) in green (n_Young_ = 8 mice, n_Aged_ = 9 mice, n_Aged+GDF11_ = 9 mice) relative to quantification Fig. 2c. Scale bar: 100 μm.

We measured levels of neurogenesis by assessing the levels of Sox2 and doublecortin (DCX). We found a 35% significant increase in Sox2^+^ NSCs in the subgranular layer (SGL) of the aged GDF11-treated DG compared to aged mice (Fig. 2a,b), and analysis of DCX revealed a 53% increase in the number of immature neuroblasts in the neurogenic SGL of GDF11-treated aged mice compared to aged controls (Fig. 2c and Extended Data Fig. 2a).

Next, we examined hippocampal senescence by quantifying SA-βGal^+^ cells in the SGZ of the DG. We found a significant twofold increase in the aged SGZ compared to the young (aged, 19.2 ± 1.7; young, 9.6 ± 1.2), but GDF11 treatment resulted in a decrease in the number of SA-βGal^+^ cells (GDF11, 11.8 ± 1.6) (Fig. 2d,e). Moreover, we quantified the expression of *Cdkn2a* (cyclin-dependent kinase inhibitor 2 A) locus transcripts encoding p16^INK4A^ and p19^ARF^, critical senescence mediators. Consistent with previous reports^28^, we observed a tenfold increase in p16^INK4A^ (Fig. 2f) and a fivefold increase in p19^ARF^ expression (Fig. 2g) with aging. Remarkably, p16^INK4A^ and p19^ARF^ expression were significantly reduced by 70% (Fig. 2f) and 30% (Fig. 2g), respectively, in GDF11-treated aged mice.

Subsequently, we analyzed the expression of key proteins involved in autophagy, the impairment of which has been linked to cognitive decline^29^. Western blot analyses of hippocampal tissues revealed significant decreases with aging in the levels of the following proteins (71% for FoxO3a, 70% for Beclin 1 and 23% for LC3 lipidation (Fig. 2i–k) respectively). Interestingly, aged mice treated systemically with GDF11 showed a significant upregulation of all the above proteins, including a significant 5.2-fold increase in FoxO3a (Fig. 2h,i), a significant 2.5-fold increase in Beclin 1 (Fig. 2h,j) and a significant 1.4-fold increase in LC3 lipidation (Fig. 2h,k).

Together, these analyses demonstrate that systemic GDF11 treatment profoundly affects cellular events in the aged brain.

### Brain infusion of GDF11 recapitulates the effects of systemic administration in a neurogenesis-independent fashion

**Extended Data Fig. 3:**
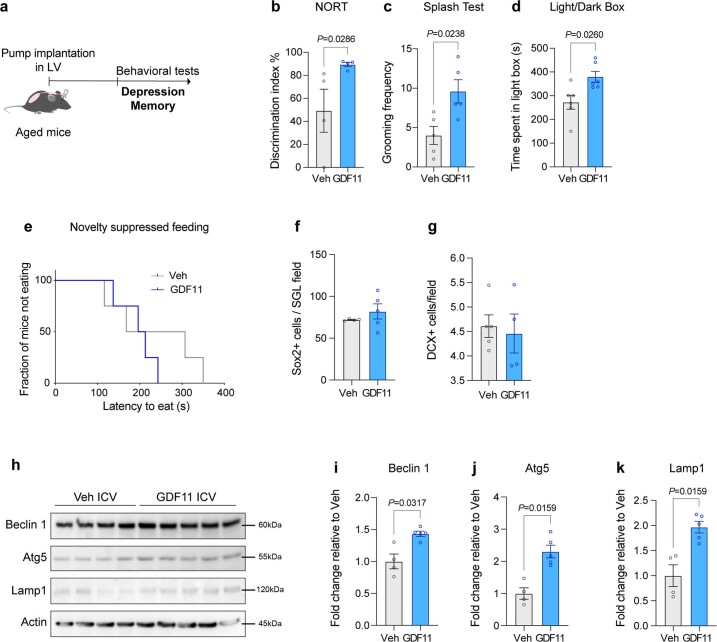
Intracerebroventricular GDF11 infusion recapitulates all aspects of systemic treatment except neurogenesis. (**a**) Schematic representation of the experimental procedure: miniosmotic pumps were implanted in the lateral ventricle and rGDF11 or vehicle was infused at a constant rate over 2 weeks. (**b**) Measurement of discrimination index during the NORT (percentage of time spent to observe the novel object divided by the percentage of total investigation time for both objects) (n = 4 mice per group). (**c**) Measurement of grooming frequency during the Splash test (n = 5 mice per group). (**d**) Measurement of time spent in the Light box during the Light/Dark Box Test (n = 6 mice per group). (**e**) Representation of latency to eat for mice during the Novelty Suppressed feeding test (n = 5 mice per group). (**f**) Quantification of Sox2^+^ NSCs in the SGL of the dentate gyrus (n_Veh_ = 4 mice, n_GDF11_ = 5 mice). (**g**) Quantification of DCX^+^ neuroblasts in the SGL of the dentate gyrus (n_Veh_ = 4 mice, n_GDF11_ = 5 mice). (**h**–**k**) Quantification of Western blots by optical density for Beclin 1 (**i**) (n_Veh_ = 4 mice, n_GDF11_ = 5 mice), Atg5 (**j**) (n_Veh_ = 4 mice, n_GDF11_ = 5 mice), and Lamp1 (**k**) (n_Veh_ = 4 mice, n_GDF11_ = 5 mice). Two-sided Mann–Whitney test for two-group comparisons; *P* values < 0.05 are represented on the graph; mean ± s.e.m. Source data

To examine the direct effect of GDF11 on NSCs and neurons, we performed brain infusions using intracerebroventricularly (ICV) implanted cannulas connected to miniosmotic pumps into the lateral brain ventricle of 21-month-old C57BL/6JRj male mice. The pumps continuously delivered rGDF11 (0.3 mg kg^−1^) or vehicle for 2 weeks (Extended Data Fig. 3a), after which memory, anxiety-like and depression-like phenotypes were analyzed by behavioral tests. In the NORT, GDF11-infused aged mice showed a significant 1.8-fold increase in the discrimination index compared to vehicle-infused mice (GDF11, 89.4 ± 1.9%; vehicle, 49.3 ± 18.6%; Extended Data Fig. 3b). In the splash test, ICV GDF11 induced a significant 2.4-fold increase in grooming frequency compared to control (GDF11, 9.6 ± 1.4; vehicle, 4 ± 1.1; Extended Data Fig. 3c). Moreover, ICV GDF11-infused mice exhibited a 1.4-fold significant increase in the time spent in the light box (GDF11, 380 ± 22.5 s; vehicle, 272 ± 29.3; Extended Data Fig. 3d). During the novelty suppressed feeding test, GDF11-infused mice exhibited shorter latency to eat compared to vehicle-infused mice (Extended Data Fig. 3e). These findings demonstrate that direct GDF11 infusion into the brain results in the same behavioral outcomes as systemic injections. Next, we examined neurogenesis and autophagy. Quantification of Sox2^+^ NSCs and DCX^+^ neuroblasts in the DG of GDF11- or vehicle-infused aged mice revealed that direct ICV infusion of GDF11 had no effect on hippocampal neurogenesis (Extended Data Fig. 3f,g, respectively). Conversely, western blot analysis of hippocampi showed a significant 1.4-fold increase in Beclin 1, a significant 2.3-fold increase in Atg5 and a significant 2-fold increase in Lamp1 (Extended Data Fig. 3h–k, respectively). Thus, direct infusion of GDF11 in the brain has distinct effects compared to systemic administration and does not affect neurogenesis.

### Treatment of NSCs with rGDF11 does not promote neuronal differentiation

**Extended Data Fig. 4:**
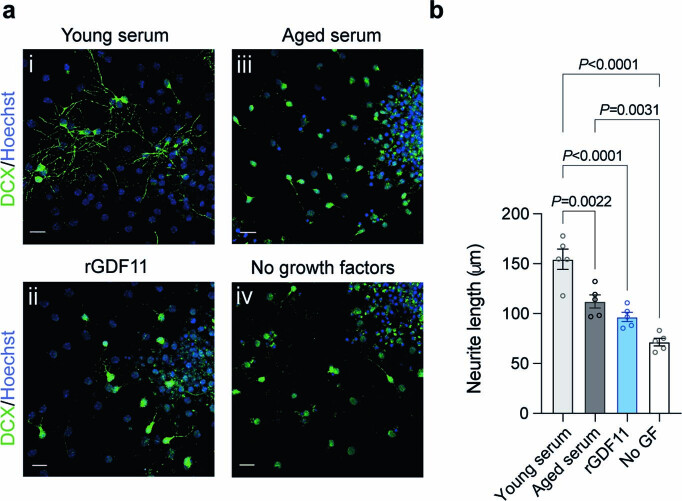
GDF11 has no effect on neurogenesis in vitro. (**a**) Representative confocal images of neural stem cell differentiation into neuroblasts upon serum treatment (4 μl), rGDF11 protein (40 ng/ml) or no serum/no growth factors in the culture medium, immunostained for DCX (green) and Hoechst (blue). (**b**) Quantification of total neurite length per neuroblast (n = 5 wells per condition; F (3, 16) = 26.4). Scale bar, 20 μm. One-way ANOVA and Tukey’s *post hoc* test for multiple comparisons; F (DFn, DFd) value presented for each ANOVA statistical analysis; *P* values < 0.05 are represented on the graph; mean ± s.e.m. Source data

To further examine the direct effect of GDF11 on NSCs and progenitor cells, we used primary cultures of neurospheres derived from 2-month-old C57BL/6JRj male mice^8,30^. Adherent neurospheres were incubated with rGDF11 (40 ng ml^−1^), and serum isolated from either young or old mice was used as a positive or negative control, respectively, as previously shown^31^. After 4 days of incubation, we measured total neurite outgrowth, a crucial process of neural stem cell differentiation and subsequent neuroblast maturation, by analyzing total neuronal process extensions. Young serum induced a 1.4-fold significant increase in neurite length compared to old serum, 1.6-fold compared to rGDF11 and 2.1-fold compared to the serum-free/growth factor-free condition (young serum, 154.5 ± 10.1; aged serum, 112.2 ± 6.6, GDF11, 96.7 ± 4.6, control:71.5 ± 3.8; Extended Data Fig. 4a,b). GDF11 treatment had the same effect as the serum-free/growth factor-free control (Extended Data Fig. 4a,b), suggesting that GDF11 does not enhance neurogenesis, in agreement with in vivo results.

Together, the above results indicate a neurogenesis-independent pathway accounting for the effects of GDF11 on both memory and depression-like behavior.

### GDF11 modulates neuronal activity and enhances autophagy in hippocampal neurons in vitro

**Fig. 3:**
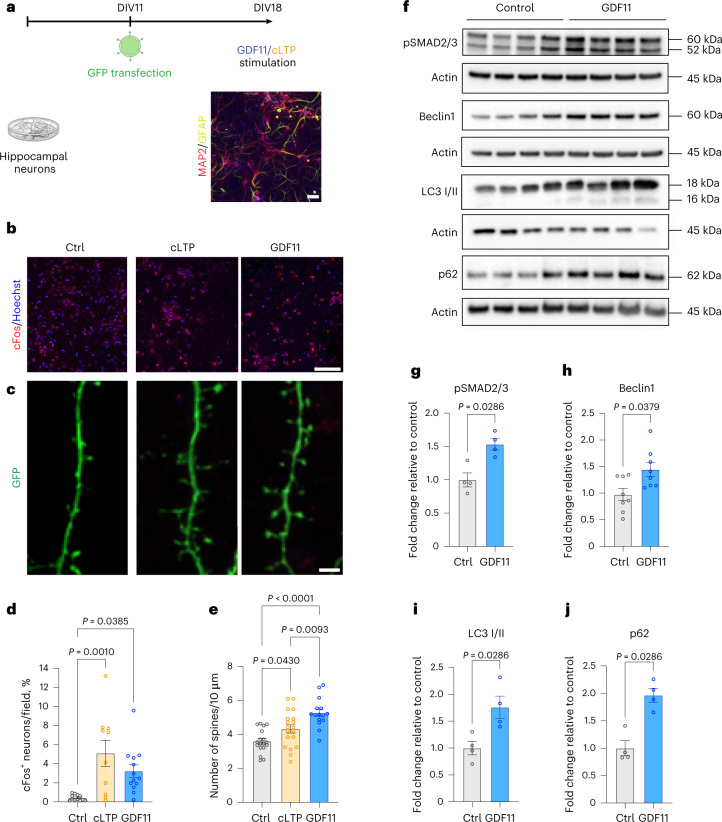
GDF11 modulates neuronal activity and enhances autophagy in hippocampal neurons in vitro. **a**, Schematic representation of the primary hippocampal neuronal cultures in vitro. Neurons were transfected with a GFP plasmid on DIV11 (only for the spine density experiment) and treated on DIV18. Scale bar: 20 μm. **b**, Representative confocal images of primary hippocampal neurons in vitro treated with vehicle (Ctrl), cLTP (positive control) or rGDF11 (40 ng ml^−1^) for 2 h on DIV18 and immunostained with cFos (red) and Hoechst (blue). Scale bar: 100 μm. **c**, Representative confocal images of GFP^+^ hippocampal neurons transfected with GFP (green) on DIV11 and treated with either vehicle (Ctrl) or cLTP (positive control) or rGDF11 (40 ng ml^−1^) on DIV18. Scale bar: 4 μm. **d**, Quantification of % cFos^+^ neurons per field (*n*_Ctrl_ = 12 fields, *n*_cLTP_ = 10 fields, *n*_GDF11_ = 13 fields; F (2, 32) = 8.3). **e**, Quantification of dendritic spine density after a 2-h stimulation with either GDF11 or cLTP or vehicle (*n*_Ctrl_ = 18, *n*_cLTP_ = 19, *n*_GDF11_ = 15 neurons examined over three independent experiments; F (2, 49) = 14.4). Numbers represent the number of spines for every 10 μm of primary dendrite. **f**, Western blots images of lysates from hippocampal neurons treated with GDF11 or control (vehicle). **g**–**j**, Quantification of western blots by optical density for phospho-SMAD2/3 (g) (*n* = 4 biologically independent samples per condition), Beclin 1 (h) (*n* = 8 biologically independent samples per condition), LC3 (i) (*n* = 4 biologically independent samples per condition) and p62 (j) (*n* = 4 biologically independent samples per condition). One-way one-sided ANOVA and Tukey’s post hoc test for multiple comparisons; two-sided Mann–Whitney test for two-group comparisons; F (DFn, DFd) values presented for each ANOVA statistical analysis; *P* values <0.05 are represented on the graph; mean ± s.e.m. Source data

Next, we sought to investigate the direct effect of GDF11 on hippocampal neurons. We established in vitro cultures of primary hippocampal neurons derived from C57BL/6JRj mouse embryonic day 15.5 (E15.5) hippocampi. As depicted in Fig. 3a, hippocampal neurons were plated on day 0 and were allowed to mature for the next 18 days in vitro (DIV). On DIV18, neurons were stimulated with either rGDF11 (40 ng ml^−1^), chemical LTP (cLTP; positive control) or vehicle for 30 min and changes in neuronal activity were assessed by immunostaining for the expression of cFos. We found that the number of neurons expressing cFos was significantly enhanced 11-fold by cLTP and 7.5-fold by GDF11, compared to control (cLTP, 5.1 ± 1.3%; GDF11, 3.2 ± 0.6%; control, 0.44 ± 0.08%; Fig. 3b,d). To assess dendritic spine density, another measure of neuronal activity, the neurons were first transfected with a GFP plasmid on DIV11 to allow subsequent visualization of spines (Fig. 1a). On DIV18, neurons were stimulated with either GDF11 (40 ng ml^−1^), cLTP or vehicle for 2 h. cLTP induced a significant increase of spine density by 23% compared to control, whereas GDF11 induced a 48% significant increase compared to control and 21% compared to cLTP (cLTP, 4.3 ± 0.2; GDF11, 5.2 ± 0.2; control, 3.6 ± 0.1; Fig. 3c,e). These results suggest that GDF11 can directly stimulate neuronal activity in hippocampal neurons, at least in vitro.

Next, we examined whether GDF11 signals via the canonical SMAD2/3 pathway. On DIV18, the neurons were treated with rGDF11 (40 ng ml^−1^) or vehicle. Western blot analysis showed enhanced SMAD2/3 phosphorylation in GDF11-treated neurons (Fig. 3f,g). Furthermore, we measured the levels of Beclin 1, which is activated at the initiation of the autophagy process; LC3I/II, which allows for the evaluation of autophagosome formation; and p62. Western blot analysis revealed that a 30-min GDF11 stimulation significantly increased the expression of Beclin 1 by 1.4-fold (Fig. 3f,h), LC3I/II by 1.7-fold (Fig. 3f,i) and p62 by 1.9-fold (Fig. 3f,j).

Taken together, our results showed that GDF11 regulates neuronal activity and its canonical SMAD2/3 signaling pathway and enhances autophagy in primary hippocampal neurons.

### GDF11 stimulation of neuronal activity is mediated by autophagy

**Fig. 4:**
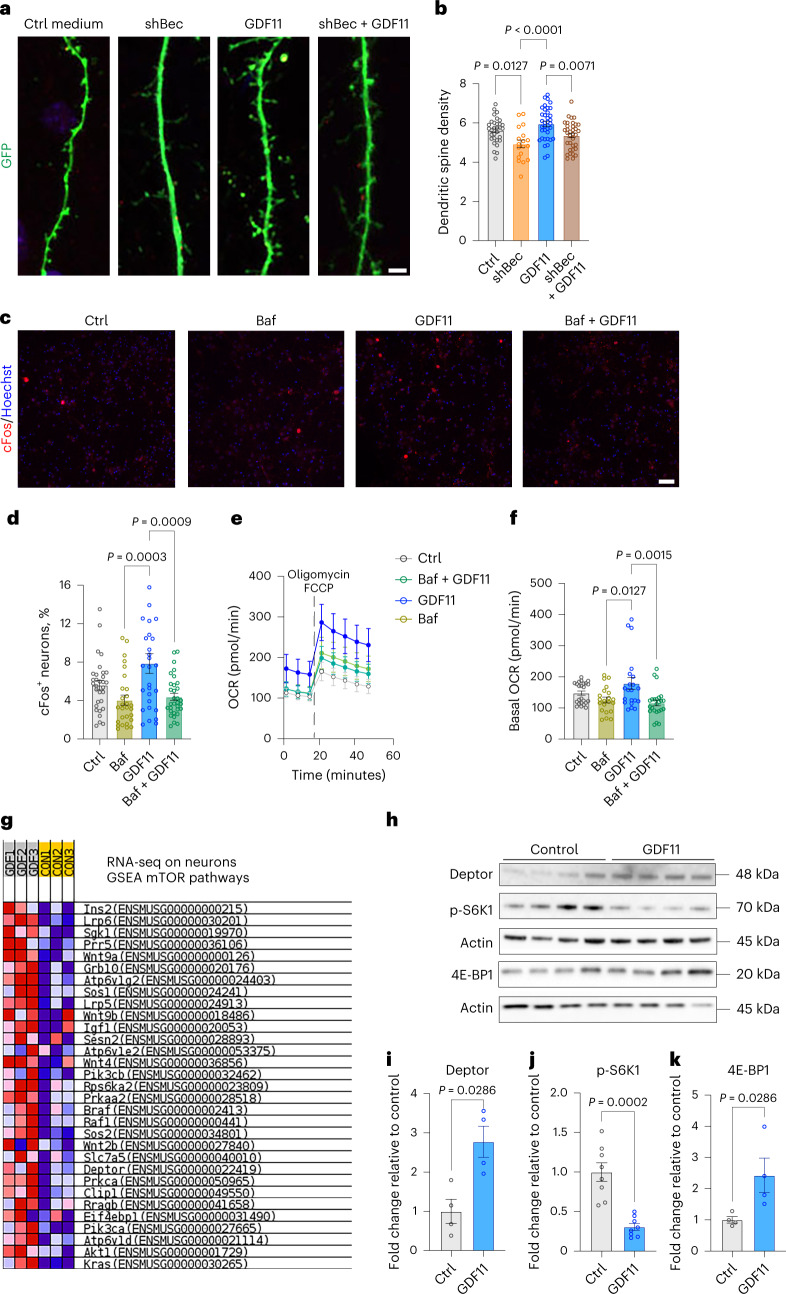
GDF11 stimulation of neuronal activity is mediated by autophagy. **a**, Representative confocal images of GFP^+^ dendrites and spines from hippocampal neurons in culture transfected with either shBeclin 1 or GFP (green) and treated with either GDF11 or control (vehicle). Scale bar: 4 μm. **b**, Quantification of dendritic spine density after a 2-h stimulation with either GDF11 or control (number represents number of spines for every 10 μm of dendrite; *n*_Ctrl_ = 31, *n*_shBec_ = 18, *n*_GDF11_ = 36, *n*_shBec+GDF11_ = 35 neurons examined over three independent experiments; F (3, 116) = 8.2). **c**, Representative confocal images of hippocampal neurons in vitro treated with vehicle, Baf or GDF11 or both and immunostained with cFos (green) and Hoechst (blue). Scale bar: 100 μm. **d**, Quantification of % cFos+ neurons per field (*n*_Ctrl_ = 31 fields, *n*_Baf_ = 28 fields, *n*_GDF11_ = 28 fields, *n*_Baf+GDF11_ = 32 fields; F (3, 115) = 7.2). **e**, Oxygen consumption rate (OCR) in primary hippocampal neurons in vitro treated for 2 h with either GDF11 or Baf or both and measured with the Seahorse analyzer (*n*_Ctrl_ = 20, *n*_Baf_ = 8, *n*_GDF11_ = 8, *n*_Baf+GDF11_ = 8 biologically independent samples). **f**, Measurement of basal OCR before oligomycin/FCCP addition (for each condition the three measurements were pooled; *n*_Ctrl_ = 24, *n*_Baf_ = 21, *n*_GDF11_ = 21, *n*_Baf+GDF11_ = 24 biologically independent samples; F (3, 86) = 5.5). **g**, GSEA from RNA-seq on neurons treated with GDF11 or vehicle for 2 h. **h**, Western blots images of lysates from hippocampal neurons treated with GDF11 or control (vehicle). **i**–**k**, Quantification of western blots by optical density for Deptor (i) (*n* = 4 biologically independent samples), phospho-S6K1 (j) (*n* = 8 biologically independent samples) and 4E-BP1 (k) (*n* = 4 biologically independent samples). Baf refers to Baf. One-way one-sided ANOVA and Tukey’s post hoc test for multiple comparisons; F (DFn, DFd) values presented for each ANOVA statistical analysis; two-sided Mann–Whitney test for two-group comparisons; *P* values < 0.05 are represented on the graph; mean ± s.e.m. Source data

To investigate whether GDF11-induced stimulation of neuronal activity was mediated through autophagy, we knocked down *Beclin 1*, which has been shown to attenuate neuronal function^29^. *Beclin 1* was silenced by transfection with an isopropyl β-D-1-thiogalactopyranoside (IPTG)-inducible short hairpin RNA (shBec) on DIV11. A GFP plasmid was used as a marker of co-transfection, and neurons were stained with anti-LacI to confirm co-transfection with the GFP. On DIV18, transfected neurons were treated with either rGDF11 (40 ng ml^−1^) or vehicle for 2 h. GDF11 treatment significantly increased spine density by 22.5% compared to shBec-silenced neurons without treatment (GDF11, 6.0 ± 0.1%; shBec, 4.9 ± 0.2%; Fig. 4a,b). However, GDF11 treatment on shBec-silenced neurons significantly reduced spine density by 10% compared to non-silenced neurons (GDF11, 6.0 ± 0.1%; shBec + GDF11, 5.4 ± 0.1%; Fig. 4a,b). We also perturbed the autophagic process by blocking autophagosome–lysosome fusion using Bafilomycin A1 (Baf). DIV18 neurons were treated with Baf (100 nM), GDF11 or both (Baf, 100 nM + GDF11 (40 ng ml^−1^)) for 2 h. cFos activation was used to evaluate neuronal activity. The decrease in neuronal autophagy by Baf resulted in a significant reduction in the number of cFos^+^ neurons (control, 5.6 ± 0.4%; Baf, 4 ± 0.5%;), whereas treatment with GDF11 increased the number of cFos^+^ neurons compared to control (Ctrl, 5.6 ± 0.4%; GDF11, 8 ± 1%; Fig. 4c,d). However, Baf treatment resulted in an attenuation of GDF11’s effect, as shown by the decrease in the number of cFos^+^ neurons in the Baf + GDF11 condition compared to the GDF11-only condition (GDF11, 8 ± 1%; Baf + GDF11, 4.4 ± 0.3%; Fig. 4c,d). These results indicate that blocking autophagy interferes with GDF11 action on hippocampal neuron activation.

Because autophagy is tightly linked to the bioenergetic function of the cell, we examined the energy phenotypes of primary hippocampal neurons treated with Baf (100 nM) and/or GDF11 (40 ng ml^−1^) using the Seahorse analyzer. Interestingly, GDF11 treatment induced an increase in the basal OCR of neurons compared to control (Fig. 4e,f). However, neurons treated with either Baf alone or in combination with GDF11 showed decreased levels of basal OCR (Fig. 4e,f). These results show that the metabolic effect of GDF11 on neuron metabolism is abrogated when autophagy and catabolic functions of lysosomes are blocked by Baf.

To gain a deeper mechanistic understanding of the signaling pathways engaged by GDF11 treatment, we performed bulk RNA sequencing (RNA-seq) on primary neurons treated with GDF11 or control. Gene set enrichment analysis (GSEA) revealed that pathways related to mTOR activity were enriched with GDF11 treatment (Fig. 4g), which is highly relevant, as the mTOR pathway is a major regulator of autophagy^32^. In particular, we identified Deptor, an inhibitor of both mTOR complexes^33^ (Fig. 4g). To confirm the RNA-seq results, we analyzed Deptor protein levels in primary hippocampal neurons treated with GDF11 (40 ng ml^−1^) or vehicle, for 2 h. Western blot analysis revealed that Deptor levels were significantly increased by 2.8-fold in GDF11-treated neurons (Fig. 4h,i). We next examined two downstream targets of mTOR, S6 Kinase 1 (S6K1) and 4E-BP1, which are positively and negatively regulated, respectively, and showed that S6K1 phosphorylation was significantly decreased by 70% (Fig. 4h,j) and that 4E-BP1 levels were significantly increased by 2.4-fold by GDF11 treatment (Fig. 4h,k). These findings indicate that GDF11 treatment reduces mTOR activity in hippocampal neurons, which could explain the increase in neuronal autophagy.

### Systemic GDF11 administration alleviates depressive-like symptoms in a preclinical murine model of depression

**Fig. 5:**
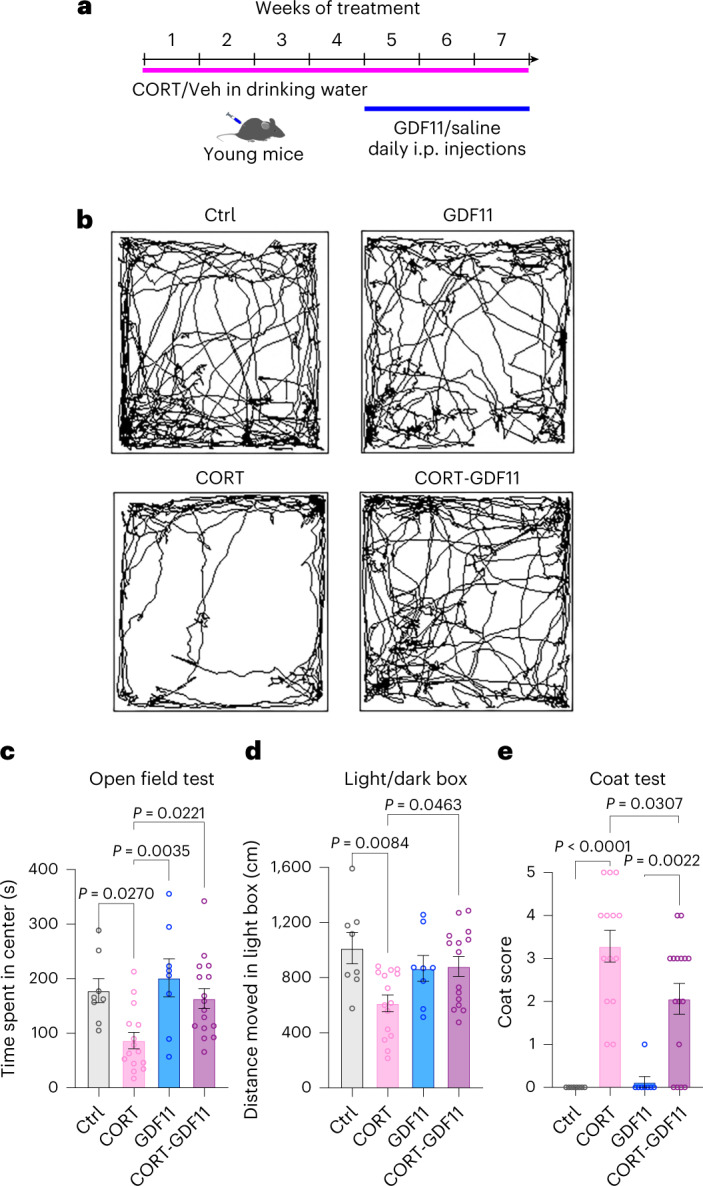
Behavioral symptoms of the depressive-like phenotype are alleviated after treatment with GDF11 in young CORT mice. **a**, Schematic representation of the experimental timeline and conditions. Veh, vehicle. **b**, Representative traces of the open field test. **c**, Measurement of the time spent in the center during the open field test (*n*_Ctrl_ = 8 mice, *n*_CORT_ = 15 mice, *n*_GDF11_ = 8 mice, *n*_CORT-GDF11_ = 16 mice; F (3, 43) = 5.9). **d**, Measurement of the distance moved in the light box during the LDB test (*n*_Ctrl_ = 8 mice, *n*_CORT_ = 15 mice, *n*_GDF11_ = 8 mice, *n*_CORT-GDF11_ = 15 mice; F (3, 42) = 4.6). **e**, Scoring of the coat state on five different areas of the mouse fur (back, abdomen, tail, forepaws and hindpaws) (*n*_Ctrl_ = 8 mice, *n*_CORT_ = 14 mice, *n*_GDF11_ = 8 mice, *n*_CORT-GDF11_ = 16 mice; F (3, 42) = 19.8). One-way ANOVA and Tukey’s post hoc test for multiple comparisons; F (DFn, DFd) values presented for each ANOVA statistical analysis; *P* values < 0.05 are represented on the graph; mean ± s.e.m. Source data

To investigate whether GDF11 could have an antidepressant effect in mice, we used a well-established preclinical model of depression-like phenotype based on chronic treatment with CORT^34^. This model produces behavioral and other alterations that resemble a limited set of features of human anxiety and depression and has previously been validated^5^. Young (2-month-old) male C57BL/6NTac male mice were given CORT in their drinking water (5 mg kg^−1^ per day), whereas control mice received vehicle (10% (2-hydroxypropyl)-beta-cyclodextrin (β-CD)). After 4 weeks of CORT or vehicle treatment, we divided these populations into two subgroups. Half of each group received daily i.p. injections of rGDF11 (1 mg kg^−1^), and the other half received saline injections (Fig. 5a), for another 3 weeks. All mice were weighed twice a week during the 7 weeks of the experiment.

**Extended Data Fig. 5:**
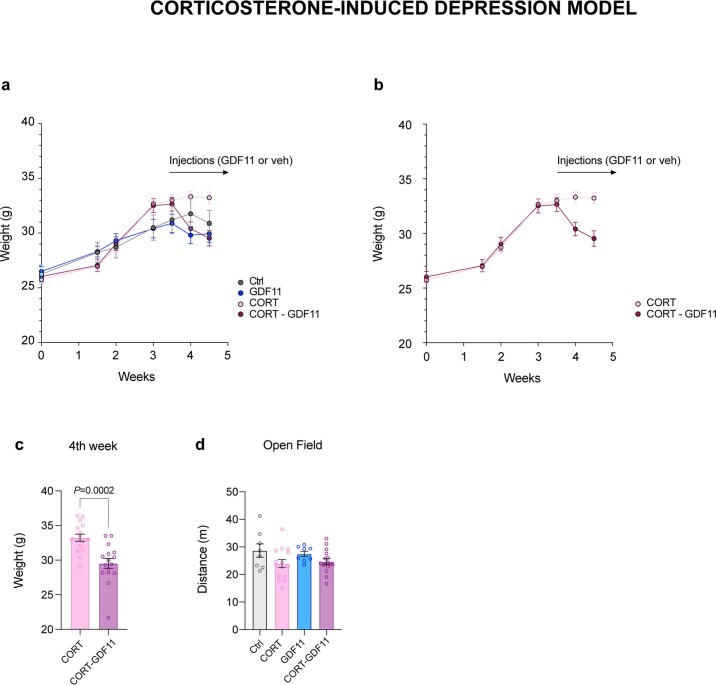
GDF11 treatment reduces weight of CORT mice. (**a**) Measurement of weight over the weeks of CORT/veh treatment and GDF11/saline injections (n_Ctrl_ = 8 mice, n_CORT_ = 16 mice, n_GDF11_ = 8 mice, n_CORT-GDF11_ = 16 mice). (**b**) Same measurement as (**a**), but only the CORT conditions are represented. (**c**) Measurement of the weight for CORT and CORT-GDF11 mice (n = 16 mice per group). (**d**) Measurement of the distance traveled during the Open Field test (n_Ctrl_ = 8 mice, n_CORT_ = 15 mice, n_GDF11_ = 8 mice, n_CORT-GDF11_ = 16 mice; F (3, 43) = 2). One-way one-sided ANOVA and Tukey’s post hoc test for multiple comparisons; F (DFn, DFd) value shown for each statistical analysis; two-sided Mann–Whitney test for two-group comparisons; *P* values < 0.05 are represented on the graph; mean ± s.e.m. Source data

After the second week of treatment, we observed a consistent weight gain for CORT mice compared to control (Extended Data Fig. 5a), which has been previously reported as a side effect of water retention due to CORT treatment^34^. Interestingly, 1 week after GDF11 injections in CORT mice, we observed a significant 11% decrease in body weight (CORT, 33.2 ± 0.5 g; CORT-GDF11, 29.5 ± 0.7 g; Extended Data Fig. 5b,c). This observation is in accordance with the previously reported role for GDF11 in inducing weight loss in mice^25,35^.

During the last 2 weeks of treatment, mice were subjected to behavioral tests to assess the effect of GDF11 on depression-like and anxiety-like phenotypes. First, we evaluated time spent in the center of the open field, which is inversely associated to anxiety-like and depression-like states (Fig. 5b). CORT mice spent 51% significantly less time in the center than control mice (Ctrl, 178 ± 22 s; CORT, 87 ± 15 s; Fig. 5c). Remarkably, CORT-GDF11 mice exhibited a significant 89% increase in time spent in the center compared to CORT mice (CORT, 87 ± 15 s; CORT-GDF11, 164 ± 18 s; Fig. 5c). No significant change was observed in the total distance traveled (Extended Data Fig. 5d), suggesting that the alleviation of the anxious-like/depressive-like phenotype was not due to possible locomotor changes. Next, we analyzed the anxiety-like state using the LDB test. CORT mice exhibited a significant 39% decrease in the distance moved in the light box compared to control mice (Ctrl, 1015 ± 113 cm; CORT, 614 ± 61 cm; Fig. 5d). On the contrary, CORT-GDF11 mice exhibited a significant 44% increase compared to CORT mice (CORT, 614 ± 61 cm; CORT-GDF11, 882 ± 72 cm; Fig. 5d), suggesting a dampening of the anxious-like phenotype. Finally, we assessed the coat state, a well-validated index of the depressive-like state^36^. A deterioration of the coat state was observed in CORT mice compared to control mice as shown by a significant increase in coat scoring, which was significantly attenuated in CORT-GDF11 mice (Ctrl, 0, CORT: 3.3 ± 0.3; GDF11, 0.1 ± 0.1; CORT-GDF11, 2.1 ± 0.3; Fig. 5e). Our findings reveal that GDF11 administration in a preclinical model of depression-like behavior was able to attenuate the depressive-like phenotype in young mice, consistent with the above results in aged mice.

### Blood levels of GDF11 inversely correlate with MDD in human subjects

**Fig. 6:**
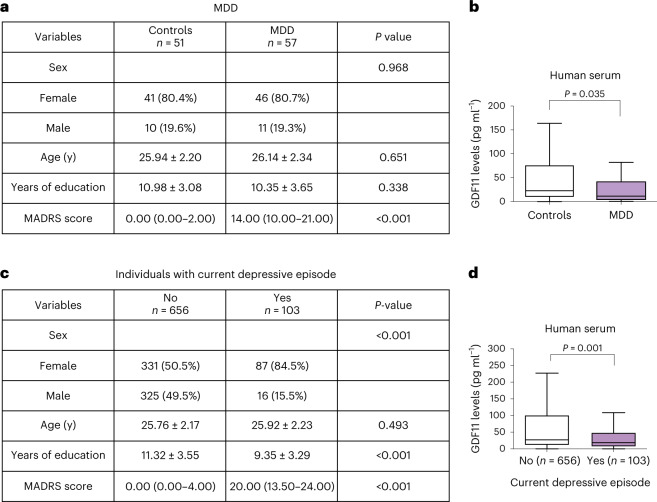
Levels of GDF11 in the blood correlate with MDD in human subjects. **a**, Table describing the clinical characteristics of the human young adults’ samples. MDD and healthy control participants were matched by sex (Pearson chi-square value = 0.002; df = 1; two-sided *P* value = 0.968), age (Student *t* test = −0.454; df = 106; two-sided *P* value = 0.651) and years of education (Student *t* test = 0.963; df = 106; two-sided *P* value = 0.338). There was a significant difference between groups in MADRS scores (Mann–Whitney *U* = 0.000; two-sided *P* value <0.001). **b**, Measurement of GDF11 by ELISA immunoassay in the serum of human healthy controls or young adults with MDD. There was a significant difference between groups in GDF11 levels (Mann–Whitney *U* = 1111.000; two-sided *P* value = 0.035). **c**, Table describing the clinical characteristics of the young adult individuals with a current depressive episode and controls. There was a significant difference between groups in sex (Pearson chi-square value = 41.613; df = 1; two-sided *P* value < 0.001), years of education (Student *t* test = 5.294; df = 752; two-sided *P* value < 0.001) and MADRS scores (Mann–Whitney *U* = 2961.500; two-sided *P* value < 0.001). There was no significant difference between groups in age (Student *t* test = −0.686; df = 757; two-sided *P* value = 0.493). **d**, Measurement of GDF11 by ELISA immunoassay in the serum of human controls or individuals with a current depressive episode. There was a significant difference between groups in GDF11 levels (Mann–Whitney *U* = 26894.000; two-sided *P* value = 0.001). The statistical analysis was conducted using the chi-squared test (sex), Student t test (age and years of education), and Mann–Whitney *U* test (MADRS and GDF11). Age and years of education are presented as mean values ± standard deviation (s.d.). MADRS are presented as the median and interquartile range. GDF11 levels are shown as Tukey boxplots, where the boxes represent the median and interquartile range. *P < 0.05. Source data

These results prompted us to explore whether blood levels of GDF11 correlate with a related pathology in humans. To address this question, we recruited participants with MDD and healthy controls of the same age and education levels. All participants were subjected to a psychiatric evaluation using the Mini International Neuropsychiatric Interview, based on the Diagnostic and Statistical Manual of Mental Disorders IV diagnostic criteria, and the Montgomery–Åsberg Depression Rating Scale (MADRS) was used to assess the severity of depressive symptoms (Fig. 6a). Blood was collected from MDD subjects and healthy age-matched controls. Serum was analyzed by enzyme-linked immunosorbent assay (ELISA) for the detection of GDF11, as previously described^35^. We first confirmed that we could exclusively detect GDF11 and not GDF8. We included young (mean age of 26) adults with MDD and healthy controls matched by sex, age, and years of education (Fig. 6a). Interestingly, serum GDF11 levels were significantly decreased in the blood of MDD subjects (n = 57), compared to healthy age-matched controls (*n* = 51) (median_CONTROL_ = 22.69 pg ml^−1^, interquartile range (IQR): 9.63–76.40), median_MDD_ = 11.66 pg ml^−1^ (IQR, 3.89–42.10); Fig. 6b). Next, we evaluated levels of GDF11 in a larger cohort of young adults who were presenting a depressive episode. This analysis included 759 young adults (103 in a current depressive episode and 656 controls) aged between 21 and 32 years old. Sample characteristics and their association with a current major depressive episode are described in Fig. 6c.

Females presented a higher prevalence of a current major depressive episode (*P* < 0.001), and individuals with a current major depressive episode had less years of education as compared to controls (*P* < 0.001). There was no significant difference between the groups regarding age.

Individuals with a current depressive episode had lower serum GDF11 levels (18.53 (IQR: 7.37–47.85)) than controls (26.97 (IQR: 11.79–100.03), *P* = 0.001). These findings reveal that blood GDF11 levels are associated with MDD as well as with a current depressive episode in humans and suggest that GDF11 could be considered as a reliable biomarker for MDD in humans.

## Discussion

We sought to determine the role of GDF11 in age-related depression-like phenotype in mice and explore the underlying mechanism. Daily systemic administration of rGDF11 resulted in an improvement of the depression-like phenotype associated with aging, as well as reversed memory decline. Likewise, direct infusion of GDF11 in the brain resulted in the same behavioral outcome. These results come in accordance with a positive effect in memory decline related to a pathological phenotype of Alzheimer’s disease, as reported in AβPP/PS1 double transgenic mice^37^. It is worth pointing out that the depressive-like phenotype and memory decline observed in aged mice is not normative. As such, aging is a risk factor for cognitive decline and depression, but only a subpopulation of individuals will present these symptoms.

We further confirmed the antidepressant role of GDF11 in a preclinical model of induced depressive-like phenotype due to chronic CORT administration and found that depressive-like symptoms were alleviated upon systemic GDF11 administration. This antidepressant effect of GDF11 in both young and old mice highlights an unknown role for GDF11 in the brain. It will be interesting to further compare the effects of GDF11 to a known antidepressant, such as fluoxetine, on possible restoration of neurogenesis and other parameters.

Regarding human pathology, we measured the levels of circulating GDF11 in the blood of young adults with MDD and found a decrease compared to healthy controls. It is crucial to note that all persons included in this study were young (average age of 26 years) and were recruited from the community to have a population-based sample. Collectively, our findings raise the possibility that serum GDF11 levels could be used as a potential biomarker to uphold the presence of depressive episodes. As these results demonstrate correlation and not causation, further clinical studies should be conducted to conclude specificity in humans. Also, it would be interesting to examine whether serum GDF11 levels are restored in MDD patients under antidepressant medication.

Given the tight links among hippocampal neurogenesis, depression and memory, we examined how GDF11 could be involved. We discovered that the increase in neurogenesis does not require longer than 9 days of GDF11 treatment and found a significant increase in both Sox2^+^ NSC populations and DCX^+^ neuroblasts in the aged hippocampus. Similar increases were reported in the hippocampus after 4 weeks of GDF11 administration^26^, suggesting that NSC activation and production of new neuroblasts is maintained at the same rate throughout the 4-week treatment. It would be interesting to examine how long this effect lasts, whether new neurons keep integrating in the DG and if there is a point in which the NSC pool would start being depleted. Examination of the NSC pool using neurospheres showed that GDF11 had no effect on neurogenesis. Moreover, direct brain infusion of GDF11 showed no increase in NSC or DCX^+^ populations in the hippocampus. These results agree with initial reports that demonstrate GDF11 taking part in a feedback inhibitory signal that limits neurogenesis in the embryonic olfactory epithelium^18,20,38^. Moreover, it was recently reported that GDF11 is a negative regulator of adult hippocampal neurogenesis by depletion of endogenous GDF11 in a tamoxifen-inducible mouse model^23^. Therefore, the observed effects on neurogenesis after systemic GDF11 administration seem to be indirect, potentially through activation of other factors in the periphery. For example, GDF11 stimulates the secretion of adiponectin from white adipose tissue^35^, and it could stimulate other peripheral molecules, such as osteocalcin. Both molecules are known to cross the blood–brain barrier and enhance neurogenesis and cognition^39–42^.

Both systemic administration and ICV infusion of GDF11 resulted in the upregulation of hippocampal autophagy. Further examination showed that GDF11 acts directly on neurons via phosphorylation of SMAD2/3 and that the autophagic process is necessary for the enhancement of neuronal activity mediated by GDF11. This comes in accordance with previous reports for GDF11 in stimulating autophagy in carotid arterial smooth muscle cells^43^ and skeletal muscle fibers^44,45^. In addition to its well-known role in lifespan extension^46^, autophagy is crucial for proper neuronal function and synaptic plasticity^47^. The fact that autophagy dysfunction leads to neurodegeneration, memory impairments and changes in mood states^47,48^, as a consequence of advanced aging, supports this idea. Likewise, the effect of GDF11 on ameliorating memory in aged mice might result from GDF11 acting as an autophagy inducer. Indeed, it was recently reported that autophagy is required for memory formation and that induction of autophagy in the brains of aged mice is sufficient to repair age-related memory decline^29^. There is some evidence that enhancement of the autophagic pathway can alleviate the depressive-like features in mice, while inhibition of Beclin 1 blocks the effects of anti-depressants^49^. Moreover, in human pathology, anti-depressants might involve modulation of autophagic pathways^50^. Mechanistically, we propose that GDF11 stimulates autophagy via inhibition of mTOR. Recently, the mTOR pathway has been shown to be compromised in the prefrontal cortex of MDD patients^51^, as well as in the murine hippocampus^52^. We provide evidence that GDF11 upregulates Deptor, an inhibitor of mTOR, and downregulates S6K1 activity, a downstream target of mTOR. Interestingly, S6K1-deficient mice exhibit a calorie restriction phenotype^53^, which resembles the effect of GDF11 in aged mice^24^. Taken together, our findings point to the conclusion that GDF11 acts as an antidepressant through modulation of autophagic pathways and mTOR.

These results reveal a connection among GDF11, mTOR, autophagy and depression and indicate that GDF11 could be considered as a reliable biomarker for MDD in humans. This role for GDF11 sheds light on its mechanism of action in the brain and allows for future therapeutic interventions in the context of depression associated with aging.

## Methods

### Human study design and participants

This paper reports the second wave of a prospective cohort study, including a population-based sample of young adults. In the first wave (2007–2009), sampling was performed by clusters, considering a population of 39 667 people in the target age range (18–24 years old), according to the current census of 448 sectors in the city Pelotas/Brazil. From these sectors, 89 census-based sectors were randomly selected. The home selection in the sectors was performed according to a systematic sampling, the first one being the house at the corner pre-established as the beginning of the sector, and the interval of selection was determined by skipping two houses. Therefore, the sample is representative of the target population due to the probabilistic sampling adopted. The first wave included 1560 young adults aged between 18 and 24 years. The second wave took place a mean of five years later (2012–2014), and all young adults included in the first wave were invited for a reassessment. Only data from the second wave are described in this current study. All participants agreed to participate in the study by providing their free and written informed consent. Compensation for travel expenses was provided for the participants. This study was approved by the Research Ethics Committee of the Universidade Católica de Pelotas under protocol number 2008/118. The full description of the study design is published elsewhere^54^.

The psychiatric diagnosis was assessed using the Mini International Neuropsychiatric Interview–Plus by trained psychologists. In addition, the severity of depressive symptoms was assessed using the MADRS.

For the first analysis using human serum in the present study, we selected 57 young adults with MDD and 51 healthy controls without mood disorders (MDD or bipolar disorder), anxiety disorders (panic disorder, agoraphobia, social phobia, generalized anxiety disorder), obsessive-compulsive disorder, post-traumatic stress disorder, or attention deficit hyperactivity disorder. The groups were matched by sex, age and years of education.

In the second analysis using human serum, we included 103 young adults presenting a current depressive episode and 656 controls without a current depressive episode.

For the GDF11 measurement, 10 ml of blood was withdrawn from each subject by venipuncture into a free-anticoagulant vacuum tube. The blood was immediately centrifuged at 3,500 *g* for 15 min, and the serum was kept frozen at −80 °C until analysis.

GDF11 serum levels were determined by sandwich-ELISA using the Human GDF-11/BMP-11 DuoSet ELISA kit according to the manufacturer’s instructions (R&D Systems) and as in Katsimpardi et al.^35^ Briefly, microtiter plates (96-well flat bottom) were coated overnight at room temperature with the anti-human GDF11 capture antibody at 4 µg ml^−1^ in PBS. Thereafter, the plates were washed three times with wash buffer and blocked with 1% BSA solution for 2 h at room temperature. After washing, the plates were incubated overnight at 4 °C with the samples and the standard curve ranged from 7.82 to 2,000 pg ml^−1^ GDF11. Plates were washed and biotinylated anti-human GDF11 detection antibody at 400 ng ml^−1^ was added, which was incubated for 2 h at room temperature. After washing, incubation with streptavidin-peroxidase conjugate (diluted 1:40 in 1% BSA solution) for 20 min at room temperature was performed and subsequently plates were washed again and incubated with the substrate solution for 20 min at room temperature. Finally, the stop solution (2 N sulfuric acid) was added and the amount of GDF11 was determined by measuring absorbance at 450 nm with correction at 540 nm. The standard curve demonstrates a direct relation between optical density and GDF11 concentration.

It is important to note that we used the GDF8 (also called myostatin) as a control for the specificity of the GDF11 kit^48^. The GDF8 (Peprotech) was tested at the same concentration as the highest GDF11 standard (2,000 pg ml^−1^) as well as at a middle value of the GDF11 standard curve (500 pg ml^−1^). GDF8 was not detected at any concentration. The statistical analysis was performed in the SPSS 21.

The distribution of numeric variables (age, years of education, MADRS and GDF11 levels) was evaluated through histograms. Numerical variables that presented normal distribution were compared between groups using Student *t* test, and numerical variables that did not present a normal distribution were compared between groups using Mann–Whitney *U* test. The dichotomous variable (sex) was compared between groups using the chi-squared test.

#### Statistics and reproducibility

No statistical method was used to predetermine sample size, because this analysis is part of a larger study; however, the sample size for these analyses is in agreement with previous literature in the field^55^. For the second analysis using human serum, we excluded from the control group (a) individuals who fulfilled criteria for a past depressive episode and were not in a current depressive episode, and (b) individuals with a history of hypomanic or manic episodes who were not in a current depressive episode. Importantly, individuals in the control group could have other psychological conditions, such as anxiety disorders. The investigators were blinded to allocation during experiments and outcome assessment.

### Animals

Young (3-month-old) and aged (22-month-old) C57BL/6JRj male mice were obtained from Janvier Labs. For the CORT experiments, 2-month-old C57BL6NTac male mice were obtained from Taconic Biosciences. All animals were group housed and provided free access to food and water. Housing conditions were as follows: dark/light cycle from 7 am to 9 pm, controlled temperature 20–24 °C, ambient humidity 60% minimum to 70% maximum. All animal procedures were performed in accordance with French legislation and in compliance with the European Communities Council Directives (2010/63/UE), according to the regulations of Institut Pasteur Animal Care Committees.

### GDF11 administration in mice

GDF11 (Peprotech, 120-11) was dissolved in water, further diluted according to the manufacturer’s instructions and injected at a concentration of 1 mg kg^−1^, as previously described in^24^. Control mice (young or aged) were injected with equivalent volumes of saline. All mice were injected daily at 7 pm for the duration of the experiment. All experimental procedures were performed in accordance to the Institut Pasteur ethical committee and the French Ministry of Research (APAFiS; 16380).

### Miniosmotic pump implantation and ICV GDF11 infusion in mice

Twenty-four hours before implantation, programmable and refillable miniosmotic pumps (iPrecio, SMP-300) were filled with saline, under sterile conditions, and programmed to deliver the infusion at a constant rate of 1 μl h^−1^. Twenty-four hours later, miniosmotic pumps were implanted into the right lateral ventricle of 21-month-old mice (antero-posterior −0.5; medial-lateral + 1.0; dorsal-ventralDV −2.0). The pumps were refilled with GDF11 solution (0.3 mg kg^−1^ rGDF11 in saline) or saline every 4 days for a total volume of 120 μl.

### Behavioral tests in mice

#### Open field test

Four gray arenas (45 × 45 cm) with high walls were used and luminosity was set at 30 lux throughout the habituation and the test phases. Mouse cages were placed in the behavior room for habituation 30 min before the test. Each mouse was placed in the arena for 10 min and then returned to their home cage.

#### NORT

Four gray arenas (45 × 45 cm) with high walls were used and luminosity was set at 30 lux throughout the habituation and the test phases. Mouse cages were placed in the behavior room for habituation 30 min before the test. Mice were habituated to the arenas for 3 consecutive days as follows. On habituation day 1, mice housed in the same cage were placed in one arena for 30 min. Then each mouse was individually placed in one arena for 10 min each. On days 2 and 3, mice were individually placed in an arena for 10 min each. On day 4 (NORT), mice were individually placed in the arena where two identical objects were placed at equal distances from the walls and from each other, and the mice were left to explore for 10 min and put back in their home cage. Two hours later, mice were placed back in the arena where one of the two objects was replaced with a novel object, and the mice were left to explore for 10 min. A camera above the arena automatically captured the locomotor activity of each mouse, and its behavioral pattern was measured and analyzed using EthoVision XT software (Noldus) and confirmed by a blind manual measurement of the time spent sniffing the objects. To exclude a spontaneous preference towards one of the objects, several pairs of objects had previously been tested with different sets of naive young and aged animals, and objects with no preference were chosen for the experiment.

#### Novel object location test

Same arenas and procedure was used as in the NORT. The test took place after the NORT, so no additional habituation was performed. The objects used were different from those in the NORT, and spatial cues were provided on the walls of the arenas (sun, moon, square and triangle shaped). On the day of the test, each mouse was individually placed in the arena where two identical objects were placed at equal distances from the walls and from each other, and the mice were left to explore for 10 min and put back in their home cage. Two hours later, mice were placed back in the arena where one of the two objects was placed in a diagonal compared to its previous position, and the mice were left to explore for 10 min. A camera above the arena automatically captured the locomotor activity of each mouse, and its behavioral pattern was measured and analyzed using EthoVision XT software (Noldus) and confirmed by a blind manual measurement of the time spent sniffing the objects. To exclude a spontaneous preference towards one of the objects, several pairs of objects had previously been tested with different sets of naive young and aged animals, and objects with no preference were chosen for the experiment.

#### Y-maze test

Mouse cages were placed in the behavior room for habituation 30 min before the experiment. A gray Y-maze arena (24.6 cm length and 7.8 cm wide) was used and luminosity was set at 30 lux throughout the training and the test phases. For the training phase, one of the arms was blocked and the mouse was placed in the center of the Y-maze to start exploring for 15 min. Then, the mouse was placed back to the home cage for 1 h, and the maze was cleaned before the next trial. For the test phase, the divider was removed and all three arms were open. The mouse was placed in the center and was left to explore the maze for 5 min. A camera above the arena automatically captured the locomotor activity of each mouse, and its behavioral pattern was measured and analyzed using EthoVision XT software (Noldus).

#### Splash test

Mouse cages were placed in the behavior room for habituation 30 min before the experiment. Then, all mice, except for the experimental mouse, were transferred to a different cage. The experimental mouse was given a spray shot of a 10% sucrose solution on the lower back and then put back in the home cage. The viscosity of the sucrose solution dirties the fur, inducing a grooming behavior. The mouse was observed for 6 min, during which the frequency of grooming, the latency to first groom and the total grooming times were measured. After the test, the experimental mouse was placed in a different cage until all the mice of the cage had been subjected to the test.

#### TST

Mouse cages were placed in the behavior room for habituation 30 min before the experiment. One mouse per condition was placed in the TST apparatus by applying tape on the mouse’s tail and using it to hang the mouse upside down. The test lasted for 6 min, during which immobility time was measured by a blind observer.

#### Sucrose preference test

Each mouse was housed individually starting in the evening of day 0. Two bottles with water were placed in each cage to habituate the mouse to the new drinking scheme. The next evening (day 1), one of the two bottles was replaced with 1% (wt/vol) solution and the other one with water. The next morning (day 2), water and sucrose-water consumption was measured. Then, the position of the bottles was changed to avoid place preference. The next morning (day 3), water and sucrose-water consumption was measured again and mice were returned to their home cages. The sucrose index we report was the average of the two measurements.

#### Social interaction test

The same arenas as for the open field test, NORT and novel object location test were used. Mouse cages were placed in the behavior room for habituation 1 h before the experiment. Each test mouse was placed in the center of the arena. Immediately after, a stimulus mouse (intruder) was also placed in the center of the arena. Mice were left to explore the arena and interact for 5 min. A camera above the arena automatically captured the locomotor activity of the mice, and their behavioral patterns were measured and analyzed using EthoVision XT software (Noldus). Active avoidance was measured manually by a blind investigator by considering a movement of the test mouse further than 3 cm away when the intruder approaches.

#### Light/Dark box test

The light box (16.5 × 22 cm) was separated from the dark box (16.5 × 22 cm) with a dark divider with a small opening allowing the mice to go through. Time spent and frequency of entry in the light box (178 lux) were measured during 10 min. A camera above the arena automatically captured the locomotor activity of the mice, and their behavioral patterns were measured and analyzed using EthoVision XT software (Noldus).

#### Coat state test

The total coat state score resulted from the sum of the score of five different body parts: neck, dorsal/ventral coat, tail, forepaws and hindpaws. For each body area, a score of 0 was given for a well-groomed coat and 1 for an unkempt coat. The measurements of the coat state were done by an experimenter blind to treatments.

#### Novelty suppressed feeding

Mice were put in new cages without any food 12 h before testing. A large white arena with high walls was covered with litter, and a food pellet was placed in the center under high brightness (100 lux). The mouse was placed in the arena, and the latency to first bite in the food pellet placed was measured (maximum of 5 min). As soon as the mouse bit the food pellet, it was removed from the arena and placed alone in its home cage for 5 min with one food pellet, which was weighed beforehand. The food pellet was weighed again after the 5-min trial, and the mouse was put in a new cage with food ad libitum. The amount of food consumed during these 5 min was measured.

#### EPM

Mouse cages were placed in the behavior room for habituation 1 h before the experiment. The apparatus consists of an elevated cross with two closed arms, two open arms and a center area (intersection between closed and open arms). The length of the open and closed arms was of 37.5 cm and the height 53 cm. To measure stress and anxiety, each mouse was placed in the center of the EPM and left to explore the arms for 6 min. A camera above the apparatus automatically captured the activity of the mouse and its behavioral pattern, as well as the time spent in the open and closed arms. Measurements and analysis were done using EthoVision XT software (Noldus).

#### Nest building test

Each mouse was housed individually starting in the evening of the testing day. Two intact cotton pellets (2 × 2 cm; 2.5 g) were placed in the cage and mice were left undisturbed overnight. The next morning each nest was assessed for two parameters: the height of the nest and the nest quality. The latter was given a score based on percentage of the nest height compared to mouse size and shape (0: material untouched; 5: cloud-like shape, material finely shredded).

#### Burrowing behavior test

Each mouse was individually housed during the experiment. Burrowing tubes (17 × 5 cm) were placed in the cage together with food pellets, all preweighed. Mice were left for 30 min to burrow by removing the pellets. The weight of the removed pellets was weighed and the burrowing behavior index was calculated as the percentage of the weight of the removed pellets divided by the initial weight.

#### Gait test

Mouse paws were painted with non-toxic, water-soluble paint using a different color for forepaws and hindpaws. Immediately after, mice were left to walk/run on a 60-cm paper-corridor. Stride length was calculated as the distance between the center of the hind paw to the center of the forepaw. Base length was calculated as the distance between left and right paw, for both forepaws and hindpaws.

### Chronic CORT administration depression model

CORT, purchased from Sigma-Aldrich, was sonicated for 2 h in a vehicle made of 10% (2-hydroxypropyl)-beta-cyclodextrin (β-CD; Sigma-Aldrich, H107) in water. After complete dissolution, the solution was added to the appropriate amount of water to reach the final concentration of 35 µg ml^−1^ CORT and 0.45% β-CD. CORT (35 µg ml^−1^, equivalent to about 5 mg kg^−1^ per day) or vehicle (0.45% β-CD) was available ad libitum in the drinking water in aluminum foil- wrapped bottles to protect it from light. All the bottles were changed every 3–4 days to prevent any possible degradation, as previously described^34^. Animals were weighed twice a week to verify the increase in body mass already described in this model^34^.

### Neural stem cell cultures

Neural stem cell cultures (neurospheres) were performed as described previously^30^. For proliferation, neurospheres were maintained in serum-free medium containing EGF and bFGF (both 20 ng ml^−1^) (Gibco human recombinant bFGF, 10612074 (Thermo Fisher Scientific); Gibco human recombinant EGF, 10628523 (Thermo Fisher Scientific)].

For the differentiation assay, neurospheres were plated on poly-lysine/laminin coated coverslips in serum-free, growth-factor-free media as described previously. To assess the effect of serum on differentiated neurospheres, we incubated the following sera for 4 days (20% of serum in culture medium, replaced every 2 days): serum from young mice (young serum), serum from 23-month-old mice (old serum). To assess the effect of the recombinant protein alone, we incubated rGDF11 (40 ng ml^−1^) for 4 days, in the same experiment as the serum assay. All incubations took place in 24-well plates. At the end of the experiment, coverslips were fixed with 4% paraformaldehyde for 30 min and then stained as described below.

### Cultures of primary hippocampal neurons

Hippocampal neurons were isolated from mouse embryos (embryonic day 16.5), as in Glatigny et al.^29^ Briefly, after dissection, hippocampi were digested with trypsin 0.05% and EDTA 0.02% for 15 min at 37 °C. After three washes with DMEM (Thermo Fisher Scientific, 61965059) supplemented with 10% FBS, 100 U ml^−1^ penicillin/streptomycin and 1x GlutaMAX (Thermo Fisher Scientific), cells were dissociated by pipetting up and down, and then plated. The dissociated cells were plated onto poly-L-lysine-coated plates or glass coverslips for microscopic examination. Twenty-four hours after plating, the media was replaced with Neurobasal medium (Thermo Fisher Scientific) containing B27 supplement (Thermo Fisher Scientific), GlutaMAX and Mycozap (Lonza). Media was changed two times per week and neurons were maintained in 5% CO2 at 37 °C. Experiments were performed on cells after 18 days of culture. Plasmid transfection was performed with Lipofectamine 2000 (Invitrogen, 11668019), according to the manufacturer’s instructions. For Beclin1 silencing experiments, neurons were transfected with shRNA targeting Beclin 1 lentivirus plasmid (pLKO-IPTG-3XLacO) expressing an IPTG-inducible shRNA targeting mouse Beclin-1 (*Becn 1*) (Sigma-Aldrich). Transfected neurons were treated with 5 mM IPTG on DIV15 and for 72 h to induce shRNA-*Beclin-1* expression. Neurons were stained with anti-LacI antibody (05-503I, Sigma-Aldrich) to confirm co-transfection with the GFP plasmid.

For neurons treated with Bafilomycin, neurons were incubated with 100 nM of Baf (B1793, Sigma-Aldrich) for 2 h before fixation. The same concentration was used for the Seahorse analysis.

### RNA-seq of primary hippocampal neurons

#### RNA isolation

Total RNA was isolated from samples using Trizol (Life Technologies) according to the manufacturer’s instructions. After adding chloroform, the samples were centrifuged at maximum speed, and the upper phase was then used for RNA cleanup using RNAeasy (Qiagen) minicolumns following the manufacturer’s instructions. Briefly, an equal volume of 70% ethanol was added to the upper phase and processed through the columns. After two washes, the RNA was eluted with water.

#### RNA-seq

RNA-seq and bioinformatic data analysis were performed by NovoGene. Briefly, messenger RNA was purified from total RNA using poly-T oligo-attached magnetic beads. After fragmentation, the first strand cDNA was synthesized using random hexamer primers, followed by second strand cDNA synthesis. The libraries were checked with Qubit and real-time PCR for quantification and bioanalyzer for size distribution detection. Quantified libraries were pooled and sequenced on an Illumina platform, and paired-end reads were generated. Raw reads were filtered to remove those containing adapters, poly-N and low-quality reads from raw data, and Q20, Q30 and guanine-cytosine (GC) were calculated. All downstream analyses were performed on clean data with high quality. Paired-end reads were aligned to the GRCm38/mm10 reference genome using Hisat2 v2.0.5, and featureCounts v1.5.0-p3 was used to count the read numbers mapped to each gene, normalized by FPKM.

#### Differential expression analysis

Differential expression analysis of two conditions/groups (two biological replicates per condition) was performed using the DESeq2 R package (1.20.0). The resulting *P* values were adjusted using the Benjamini and Hochberg’s approach, and genes with an adjusted *P* value ≤ 0.05 found by DESeq2 were assigned as differentially expressed.

#### Enrichment analysis of differentially expressed genes

Gene Ontology enrichment analysis of differentially expressed genes was done using the clusterProfiler R package for which gene length bias was corrected. Genes were ranked according to the degree of differential expression, and then predefined gene sets were tested to using a local version of the GSEA analysis tool (http://www.broadinstitute.org/gsea/index.jsp).

### Seahorse metabolic analysis

For metabolic analyses, the Seahorse XF Cell Energy Phenotype test kit (Agilent) was used. Primary hippocampal neurons were isolated as described above and plated directly on poly-L-lysine-coated 96-well plates provided in the kit by the manufacturer. Cells were treated for 2 h before measurement of their basal OCR, and then oligomycin and FCCP were injected to induce maximal respiration in the neurons.

### Immunohistochemistry

Mice were anesthetized with a mix of ketamine (80–100 mg kg^−1^) and xylazine (10–12.5 mg kg^−1^), and their brains were removed and fixed overnight in 4% PFA. Each brain was embedded in 4% agarose, and 40-μm-thick coronal sections were cut using a vibrating microtome (VT1000S, Leica). For neurosphere cultures and primary neurons, cells were fixed on poly-L-lysine-coated coverslips. Tissue sections or cells were pre-incubated in 10% normal goat or donkey serum, 0.1% Triton-X 100 in PBS for 1 h and were incubated overnight at 4 °C with the following antibodies: rabbit polyclonal anti-Sox2 (1:100, Cell Signaling Technology, #2748), chicken polyclonal anti-DCX (1:400, abcam, ab153668), cFos (1:2000, ABE457, Millipore). Alexa Fluor (Life Technologies) secondary antibodies were used for detection of the primary antibody at a dilution of 1:1,000 (Thermo Fisher Scientific, A-21244; goat polyclonal anti-Rabbit IgG (H + L) Cross-Adsorbed Secondary Antibody, Alexa Fluor 647, lot 2086678), Thermo Fisher, A-11039, Goat polyclonal anti-Chicken IgY (H + L) Secondary Antibody, Alexa Fluor 488, lot 2180688; Merck Millipore, AP180SA6, Donkey polyclonal anti-Goat IgG (H + L) secondary antibody, Alexa Fluor 647, lot 3743391; Jackson Immunoresearch, 703-545-155, Donkey polyclonal Anti-Chicken IgY (IgG) (H + L) secondary antibody Alexa Fluor 488 AffiniPure, lot 151980; Thermo Fisher Scientific, A-21235, Goat polyclonal anti-Mouse IgG (H + L), cross-adsorbed secondary antibody, Alexa Fluor 647.

### SA-βGal assay

Mice were anesthetized with a mix of ketamine (80–100 mg kg^−1^) and xylazine (10–12.5 mg kg^−1^), and brains were perfused with 4% PFA and subsequently post-fixed in 4% PFA overnight. The following day, brains were washed three times for 15 min with PBS. Each brain was embedded in 4% agarose, and 40-μm-thick coronal sections were cut using a vibrating microtome (VT1000S, Leica). Then, the SGZ sections were selected and incubated in X-Gal solution containing 40 mM citrate buffer (pH=6) (C7129, Sigma-Aldrich), 5 mM K3Fe (CN)6 (P8131, Sigma-Aldrich), 5 mM K4Fe (CN)6 (P9387, Sigma-Aldrich), 2 mM MgCl_2_ (208337, Sigma-Aldrich), 150 mM NaCl (31434, Sigma-Aldrich) and 1 mg ml^−1^ 5-bromo-4-chloro-3-indolyl-beta-D-galactopyranoside (X-Gal) (10703729001, Sigma-Aldrich) in PBS (pH 6)] at 37 °C in the dark for 48 h. After incubation, the SGZ sections were post-fixed with 4% PFA for 15 min and then washed three times for 15 min with PBS. Finally, the sections were mounted on slides using Fluoromount-G (00-4958-02, Thermo Fisher Scientific) and observed under an optical microscope for the development of blue color referring to senescent cells. The images were taken with a scanner (Olympus VS120) and quantification of senescent cells was performed manually by a blinded investigator.

### Western blots

Hippocampi were dissected and snap frozen in liquid nitrogen. Cell cultures were spun down, the medium was removed and the pellet was snap frozen in liquid nitrogen. Tissues and cell pellets were lysed in RIPA lysis buffer (25 mM Tris-HCl pH 7.6, 150 mM NaCl, 1% NP-40, 1% sodium deoxycholate, 0.1% SDS) (Pierce, Thermo Fisher Scientific) and protease (cOmplete, Sigma-Aldrich) and phosphatase (phosSTOP, Sigma-Aldrich) inhibitors. Protein concentration was measured with the Pierce BCA protein Assay Kit. Tissue lysates were mixed with 4 × NuPage LDS loading buffer (Invitrogen), and proteins were separated on a 4–12% SDS-polyacrylamide gradient gel (Invitrogen) and subsequently transferred by semi-dry or liquid transfer onto a polyvinylidene fluoride membrane (Trans-blot Turbo Mini PVDF, Bio-Rad). The blots were blocked in 5% BSA in Tris-buffered saline with Tween (TBS**-**T) and incubated with: anti-Lamp1 (1:1,000, ab24170 abcam), rabbit anti-LC3 (1:1,000, L7543, Sigma-Aldrich), rabbit anti-Beclin1 (1:1,000, 3495, Cell Signaling), rabbit anti-Atg5 (1:1,000, 12994, Cell Signaling), rabbit polyclonal anti-FOXO3a (1:1,000, 2497, Cell Signaling Technology), rabbit polyclonal anti-total-Smad2/3 (1:1000, #13820, Cell Signaling), rabbit polyclonal anti-phospho-Smad2/3 (1:1,000, 8685, Cell Signaling), rabbit polyclonal anti-phospho-S6K1 (1:1,000, 9205, Cell Signaling), rabbit polyclonal anti-Deptor (1:1,000, Cat# ABS222, Sigma-Aldrich), rabbit polyclonal anti-phospho-4E-BP1 (1:1,000, 2855, Cell Signaling) and mouse anti-actin (1:6,000, A5441, Sigma-Aldrich). To detect protein signals, the following horseradish peroxidase (HRP)-conjugated secondary antibodies were used: Goat Anti-Rabbit IgG (H + L)-HRP conjugate (1:6,000, #1706515, Bio-Rad) and Goat Anti-Mouse IgG1 heavy chain (HRP) (1:6000, ab97240, Abcam). Chemiluminescence detection of proteins was performed with Luminata Crescendo Western HRP Substrate (Merck Millipore) and imaged with the Chemidoc Imaging System (Bio-Rad). Bands were quantified using Fiji (ImageJ) software.

### Real-time qPCR

Total RNA was extracted from tissue samples using the NucleoSpin RNA/Protein kit (NucleoSpin^®^ RNA/Protein, Macherey-Nagel, 740933) following the manufacturer’s recommendations. Samples were treated with DNase I before reverse transcription into cDNA. One microgram of RNA was reverse-transcribed with the High-Capacity cDNA Reverse Transcription Kit (Thermo Fisher Scientific, 4368813) following the provider’s recommendations and diluted fivefold with water before using for quantitative PCR. Quantitative PCR was performed using of the SYBR Green Master Mix (Roche Applied Science) on a LightCycler 480 Real-Time PCR System (Roche Applied Science, 4368708). Expression values were obtained using the 2^−ΔΔCt^ method, as previously described^56^. All assay were performed in triplicate and were normalized to *Gapdh* levels. Primers used for qPCR analysis as follows: mp16 forward, 5′-CGTACCCCGATTCAGGTGAT-3′; mp16 reverse, 5′-TTGAGCAGAAGAGCTGCTACGT-3′; mARF forward (p19), 5′-GCCGCACCGGAATCCT-3′; mARF reverse (p19), 5′-TTGAGCAGAAGAGCTGCTACGT-3′.

### Image acquisition and analysis/quantification

Imaging was performed using a Zeiss LSM 510 inverted confocal microscope, and a Zeiss Apotome microscope. Sox2 quantification was performed by batch analysis using Icy software (http://icy.bioimageanalysis.org/). Numbers of DCX^+^ cells in the DG were blindly quantified by hand, using every other section and the same area for each condition. Neurite length of neurospheres was measured with IMARIS software (Bitplane).

### Statistics and reproducibility

No statistical methods were used to predetermine sample sizes. Sample size was determined in accordance with standard practices in the field and based on our previous analyses and experience in these experimental paradigms^8,24,31^. No data were excluded from the analysis. Samples, mice and mouse cages were randomly allocated to experimental groups. Experiments were carried out in a blinded fashion; investigators were blinded during experimental procedures, data collection and data analysis by assigning codes (prepared by other investigators irrelevant to this study) to mice, mouse cages, cell samples and images before processing, to ensure unbiased analysis. Statistical tests for each experiment are mentioned in the corresponding figure legends.

All statistical analyses (except for RNA-seq) were performed using GraphPad Prism (version 9), with one-way ANOVA and Tukey’s post hoc test for multiple group comparisons and two-sided Mann–Whitney test for two-group comparisons, assuming a two-tailed distribution. Statistical significance was assigned for *P* < 0.05; results are shown as s.e.m.

Data distribution was assumed to be normal, but this was not formally tested.

### Reporting summary

Further information on research design is available in the Nature Portfolio Reporting Summary linked to this article.

## Supplementary information

Reporting Summary

## Data Availability

The primary neuron RNA-seq datasets are publicly available at the Sequence Read Archive (https://www.ncbi.nlm.nih.gov/sra/PRJNA913014; accession number PRJNA913014). Source files are available online, and all data are available from the corresponding authors upon reasonable request.

## Source data

Source Data Fig. 1 Statistical Source Data
Source Data Fig. 2 Statistical Source Data
Source Data Fig. 2 Unprocessed western blots
Source Data Fig. 3 Statistical Source Data
Source Data Fig. 3 Unprocessed western blots
Source Data Fig. 4 Statistical Source Data
Source Data Fig. 4 Unprocessed Western blots
Source Data Fig. 5 Statistical Source Data
Source Data Fig. 6 Statistical Source Data
Source Data Extended Data Fig. 1 Statistical Source Data
Source Data Extended Data Fig. 3 Statistical Source Data
Source Data Extended Data Fig. 3 Unprocessed western blots
Source Data Extended Data Fig. 4 Statistical Source Data
Source Data Extended Data Fig. 6 Statistical Source Data
