# Author Correction: Systemic GDF11 attenuates depression-like phenotype in aged mice via stimulation of neuronal autophagy

**DOI:** 10.1038/s43587-023-00454-6

**Published:** 2023-06-16

**Authors:** Carine Moigneu, Soumia Abdellaoui, Mariana Ramos-Brossier, Bianca Pfaffenseller, Bianca Wollenhaupt-Aguiar, Taiane de Azevedo Cardoso, Claire Camus, Aurélie Chiche, Nicolas Kuperwasser, Ricardo Azevedo da Silva, Fernanda Pedrotti Moreira, Han Li, Franck Oury, Flávio Kapczinski, Pierre-Marie Lledo, Lida Katsimpardi

**Affiliations:** 1 Perception and Memory Lab, Institut Pasteur, Université Paris Cité, CNRS UMR3571, Paris, France; 2 Institut Necker Enfants Malades, INSERM UMR-S1151, Université Paris Cité, Paris, France; 3 Department of Psychiatry and Behavioural Neurosciences, McMaster University, Hamilton, ON Canada; 4 Cellular Plasticity in Age-Related Pathologies Laboratory, Institut Pasteur, Université Paris Cité, CNRS UMR3738, Paris, France; 5 Department of Health and Behavior, Catholic University of Pelotas, Pelotas, Brazil; 6 Instituto Nacional de Ciência e Tecnologia Translacional em Medicina (INCT-TM), Porto Alegre, Brazil; 7 Department of Psychiatry, Universidade Federal do Rio Grande do Sul (UFRGS), Porto Alegre, Brazil; 8 Present Address: Institut Necker Enfants Malades, INSERM UMR-S1151, Université Paris Cité, Paris, France

**Keywords:** Neuroscience, Cognitive ageing

Correction to: *Nature Aging* 10.1038/s43587-022-00352-3. Published online 2 February 2023.

In the version of this article initially published, author Claire Camus (Perception and Memory Lab, Institut Pasteur, Université Paris Cité, CNRS UMR3571, Paris, France) was mistakenly omitted from the author list. In addition, the Acknowledgements section omitted a grant from the Ontario Research Fund – Ministry of Research and Innovation, the Canada Foundation for Innovation John Evans Leaders Fund (CFI-JELF 37667). The errors have been corrected in the HTML and PDF versions of the article.

