# Supplementary material for: Systemic GDF11 attenuates depression-like phenotype in aged mice via stimulation of neuronal autophagy
Source: Nat Aging. 2023 Feb 2;3(2):213–28. doi: 10.1038/s43587-022-00352-3 (PMC10154197; doi:10.1038/s43587-022-00352-3)
Supplement: Supplementary file 1 — Reporting Summary [file 43587_2022_352_MOESM1_ESM.pdf]

nature portfolio

Corresponding author(s): Dr. Lida Katsimpardi
Last updated by author(s): Dec 3, 2022

Reporting Summary

Nature Portfolio wishes to improve the reproducibility of the work that we publish. This form provides structure for consistency and transparency in reporting. For further information on Nature Portfolio policies, see our Editorial Policies and the Editorial Policy Checklist.

Please do not complete any field with "not applicable" or n/a. Refer to the help text for what text to use if an item is not relevant to your study.
For final submission: please carefully check your responses for accuracy; you will not be able to make changes later.

Statistics

For all statistical analyses, confirm that the following items are present in the figure legend, table legend, main text, or Methods section.

n/a	Confirmed	
☐	☒	The exact sample size (*n*) for each experimental group/condition, given as a discrete number and unit of measurement
☐	☒	A statement on whether measurements were taken from distinct samples or whether the same sample was measured repeatedly
☐	☒	The statistical test(s) used AND whether they are one- or two-sided<br>*Only common tests should be described solely by name; describe more complex techniques in the Methods section.*
☐	☒	A description of all covariates tested
☐	☒	A description of any assumptions or corrections, such as tests of normality and adjustment for multiple comparisons
☐	☒	A full description of the statistical parameters including central tendency (e.g. means) or other basic estimates (e.g. regression coefficient) AND variation (e.g. standard deviation) or associated estimates of uncertainty (e.g. confidence intervals)
☐	☒	For null hypothesis testing, the test statistic (e.g. *F*, *t*, *r*) with confidence intervals, effect sizes, degrees of freedom and *P* value noted<br>*Give P values as exact values whenever suitable.*
☒	☐	For Bayesian analysis, information on the choice of priors and Markov chain Monte Carlo settings
☒	☐	For hierarchical and complex designs, identification of the appropriate level for tests and full reporting of outcomes
☒	☐	Estimates of effect sizes (e.g. Cohen's *d*, Pearson's *r*), indicating how they were calculated

Our web collection on statistics for biologists contains articles on many of the points above.

Software and code

Policy information about availability of computer code

Data collection	This study did not generate new codes. The following commercial software packages were used for data collection:<br>Behavioral experiments: Ethovision Noldus (https://www.noldus.com/ethovision-xt)<br>Confocal microscopy: Zeiss 980 Airyscan2 (https://www.zeiss.com/microscopy/en/products/light-microscopes/confocal-microscopes/lsm-980-with-airyscan-2.html)<br>Microscopy: Olympus VS120 (https://www.olympus-lifescience.com/en/microscopes/virtual/vs120/)<br>Microscopy: Zeiss Apotome 3 (https://www.zeiss.com/microscopy/en/products/light-microscopes/widefield-microscopes/apotome-3.html)<br>qPCR data acquisition: LightCycler 480 Real-Time PCR System, Roche (https://diagnostics.roche.com/global/en/products/instruments/lightcycler-480-ins-445.html)<br>Western blots: Chemidoc, Biorad (https://www.bio-rad.com/fr-fr/category/chemidoc-imaging-systems?ID=NINJ0Z15)<br>RNA-Sequencing performed by Novogene (https://en.novogene.com/): Illumina platform,Hisat2 v2.0.5 and featureCounts v1.5.0-p3<br>Seahorse metabolic phenotyping: Seahorse XF Pro Analyzer (https://www.agilent.com/en/product/cell-analysis/real-time-cell-metabolic-analysis/xf-analyzers/seahorse-xf-pro-analyzer-1980223)<br>ELISA immunoassay: iMark microplate reader, Biorad (https://www.bio-rad.com/fr-fr/product/imark-microplate-absorbance-reader?ID=ada40399-b5fe-4917-8423-377a3e0c3b44)
Data analysis	This study did not generate new codes. The following commercial software packages were used for data analysis:<br>Statistical analysis and generation of graphs: GraphPad Prism 9.4.1 (https://www.graphpad.com/scientific-software/prism/) or R software, with one-way ANOVA and Tukey's post hoc test for multiple group comparisons and Mann-Whitney test for two-group comparisons, assuming a two-tailed distribution. Statistical significance was assigned for p<0.05; results are shown as standard error of the mean (S.E.M.).<br>Behavioral ex<br>periments: Ethovision XT software Noldus (https://www.noldus.com/ethovision-xt)

Quantification and image analysis: Fiji (https://imagej.net/software/fiji/), Imaris (https://imaris.oxinst.com/imaris-viewer) and Icy (https://icy.bioimageanalysis.org/) softwares
Seahorse metabolic phenotyping: Seahorse XF Pro Analyzer (https://www.agilent.com/en/product/cell-analysis/real-time-cell-metabolic-analysis/xf-analyzers/seahorse-xf-pro-analyzer-1980223)
RNA-Sequencing performed by Novogene (https://en.novogene.com/): R package DESeq2 R package (1.20.0) and GSEA analysis tool (http://www.broadinstitute.org/gsea/index.jsp).

For manuscripts utilizing custom algorithms or software that are central to the research but not yet described in published literature, software must be made available to editors and reviewers. We strongly encourage code deposition in a community repository (e.g. GitHub). See the Nature Portfolio guidelines for submitting code & software for further information.

Data

Policy information about availability of data

All manuscripts must include a data availability statement. This statement should provide the following information, where applicable:
- Accession codes, unique identifiers, or web links for publicly available datasets
- A description of any restrictions on data availability
- For clinical datasets or third party data, please ensure that the statement adheres to our policy

The primary neuron RNA-sequencing datasets are publicly available at Zenodo (DOI: 10.5281/zenodo.7389272). Source files are available online and all data are available from the corresponding authors upon reasonable request.

Human research participants

Policy information about studies involving human research participants and Sex and Gender in Research.

Reporting on sex and gender	In this population-based sample of young adults sex was considered and we have presented the analysis based on sex differences.
Population characteristics	In this population-based sample of young adults (18-24 years-old), the psychiatric diagnosis was assessed using the Mini International Neuropsychiatric Interview – Plus (MINI-Plus) by trained psychologists. In addition, the severity of depressive symptoms was assessed using the Montgomery–Åsberg Depression Rating Scale (MADRS).<br>For the first analysis using human serum in the present study, we selected 57 young adults with Major Depressive Disorder (MDD) and 51 healthy controls without mood disorders (MDD or bipolar disorder), anxiety disorders (panic disorder, agoraphobia, social phobia, generalized anxiety disorder), Obsessive-Compulsive Disorder, Post-traumatic Stress Disorder, or Attention deficit hyperactivity disorder. The groups were matched by sex, age, and years of education.<br>In the second analysis using human serum, we included 103 young adults presenting a current depressive episode and 656 controls without a current depressive episode. We excluded from the control group (a) individuals who fulfilled criteria for a past depressive episode and were not in a current depressive episode, and (b) individuals with a history of hypomanic or manic episodes who were not in a current depressive episode. Importantly, individuals in the control group could have other psychological conditions, such as anxiety disorders.
Recruitment	This is the second wave of a prospective cohort study, including a population-based sample of young adults. In the first wave (2007-2009), sampling was performed by clusters, considering a population of 39 667 people in the target age range (18-24 years-old), according to the current census of 448 sectors in the city Pelotas/Brazil. From these sectors, 89 census-based sectors were randomly selected. The home selection in the sectors was performed according to a systematic sampling, the first one being the house at the corner pre-established as the beginning of the sector, and the interval of selection was determined by skipping two houses. Therefore, the sample is representative of the target population due to the probabilistic sampling adopted. Although this sample is representative of the young adult population from the city of Pelotas in Brazil, our results may not be generalizable for other age range groups (i.e. older populations). The first wave included 1560 young adults aged between 18-24. The second wave took place a mean of five years later (2012-2014), and all young adults included in the first wave were invited for a reassessment. Only data from the second wave are described in this current study. All participants agreed to participate in the study by providing their free and informed consent.
Ethics oversight	This study was approved by the Research Ethics Committee of the Universidade Católica de Pelotas (UCPel) under protocol number 2008/118.

Note that full information on the approval of the study protocol must also be provided in the manuscript.

Field-specific reporting

Please select the one below that is the best fit for your research. If you are not sure, read the appropriate sections before making your selection.

☒ Life sciences ☐ Behavioural & social sciences ☐ Ecological, evolutionary & environmental sciences

For a reference copy of the document with all sections, see nature.com/documents/nr-reporting-summary-flat.pdf

Life sciences study design

All studies must disclose on these points even when the disclosure is negative.

Sample size	Sample size was determined in accordance with standard practices in the field and based on our previous analyses and experience in these experimental paradigms (PMID: 32187541, 31637864, 24797482).
Data exclusions	No data were excluded from the analysis.
Replication	Reproducibility of experimental findings was assured by repeating three times independent experiments, as explained in the text.
Randomization	Samples, mice and mouse cages were randomly allocated to experimental groups.
Blinding	Investigators were blinded during experimental procedures, data collection and data analysis by assigning codes (prepared by investigators irrelevant to this study) to mice, mouse cages, cell samples and images before processing, to ensure unbiased analysis.

Reporting for specific materials, systems and methods

We require information from authors about some types of materials, experimental systems and methods used in many studies. Here, indicate whether each material, system or method listed is relevant to your study. If you are not sure if a list item applies to your research, read the appropriate section before selecting a response.

Materials & experimental systems

n/a	Involved in the study	
☐	☒	Antibodies
☒	☐	Eukaryotic cell lines
☒	☐	Palaeontology and archaeology
☐	☒	Animals and other organisms
☐	☒	Clinical data
☒	☐	Dual use research of concern

Methods

n/a	Involved in the study	
☒	☐	ChIP-seq
☒	☐	Flow cytometry
☒	☐	MRI-based neuroimaging

Antibodies

Antibodies used

Immunofluorescence
Primary antibodies
Cell signaling technology, Cat#2748S, Rabbit polyclonal anti-Sox2, lot: 2
Abcam, Cat#ab153668, Chicken polyclonal anti-Doublecortin, lot: GR3334644-1
Merckmillipore, Cat#ABE457, Rabbit polyclonal anti-cFos, lot: 3168266
Abcam, Cat#ab4674, Chicken polyclonal anti-GFAP, lot: GR3424848-1
Santa Cruz Biotechnology, Cat#sc-5359, Goat polyclonal anti- MAP-2 (D-19), lot: D152
LacI: Sigma-Aldrich, Cat#05-503I, Mouse Monoclonal anti-LacI, Clone 9A5
Secondary antibodies
ThermoFisher, Cat#A-21244, Goat polyclonal anti-Rabbit IgG (H+L) Cross-Adsorbed Secondary Antibody, Alexa Fluor™ 647, Lot: 2086678
ThermoFisher, Cat#A-11039, Goat polyclonal anti-Chicken IgY (H+L) Secondary Antibody, Alexa Fluor™ 488, Lot: 2180688
Merckmillipore, Cat#AP180SA6, Donkey polyclonal anti-Goat IgG (H+L) Secondary Antibody, Alexa Fluor® 647, Lot: 3743391
Jacksonimmuno, Cat#703-545-155, Donkey polyclonal Anti-Chicken IgY (IgG) (H+L) Secondary Antibody Alexa Fluor® 488 AffiniPure, Lot: 151980
ThermoFisher, Cat#A-21235, Goat polyclonal anti-Mouse IgG (H+L), Cross-Adsorbed Secondary Antibody, Alexa Fluor, 647
Western blot
Primary antibodies
LAMP1: Abcam, Cat#ab24170S, Rabbit polyclonal anti-LAMP1
LC3B: Sigmaaldrich, Cat#L7543S, Rabbit polyclonal anti-LC3B
Beclin-1 (D40C5): Cell signaling technology, Cat#3495S, Rabbit polyclonal anti- Beclin-1 (D40C5), lot: 6
Atg5 : Cell signaling technology, Cat#12994S,Rabbit polyclonal anti- Atg5, lot: 4
FoxO3a (75D8): Cell signaling technology, Cat#2497S, Rabbit polyclonal anti- FoxO3a (75D8), lot : 6
Smad2/3 (D7G7): Cell signaling technology, Cat#8685S, Rabbit polyclonal anti- Smad2/3 (D7G7), lot : 6
Phospho-p70 S6 Kinase (Thr389) : Cell signaling technology, Cat#9205S, Rabbit polyclonal anti- Phospho-p70 S6 Kinase (Thr389)
DEPTOR : Merckmillipore, Cat#3495S, Rabbit polyclonal anti-DEPTOR
Phospho-4E-BP1 (Thr37/46) (236B4): Cell signaling technology, Cat#2855, Rabbit polyclonal anti-Phospho-4E-BP1 (Thr37/46) (236B4)
p62/SQSTM1 : Abnova, Cat#H00008878-M01, Mouse Monoclonal anti- p62/SQSTM1, Clone 2C11, lot : I2271-2C11
Actin: Sigma-Aldrich, Cat# A5441, Mouse Monoclonal Anti-b-Actin, Clone AC-15
Secondary antibodies
Bio-rad, Cat#1706515, Goat Polyclonal anti-Rabbit IgG-HRP secondary antibody conjugate,

	Abcam, Cat#ab97240, Goat Polyclonal anti-Mouse IgG1 heavy chain-HRP secondary antibody,
Validation	Antibodies used for immunostaining of brain tissue or cells correctly stained the subcellular localization of the protein as in previous reports (PMID: 31637864, 30341181, 24797482, 19911428, 18499894). Antibodies used for Western blots (phospho- or total proteins) were validated using appropriate kinase inhibitors or knockout/overexpression experiments.<br><br>Additional information for each antibody can be found on the manufacturer's website as follows:<br>rabbit polyclonal anti-Sox2 (1:100, Cell Signaling Technology, #2748), https://www.cellsignal.com/products/primary-antibodies/sox2-antibody/2748<br>chicken polyclonal anti-doublecortin (DCX) (1:400, abcam, ab153668), https://www.abcam.com/doublecortin-antibody-ab153668.html<br>rabbit polyclonal anti-cFos (1:2000, ABE457, Millipore), https://www.merckmillipore.com/FR/fr/product/Anti-c-Fos-Antibody,MM_NF-ABE457<br>rabbit polyclonalanti-Lamp1 (1:1000, ab24170 abcam), https://www.abcam.com/lamp1-antibody-lysosome-marker-ab24170.html<br>rabbit polyclonalanti-LC3 (1:1000, L7543, Sigma), https://www.sigmaaldrich.com/FR/fr/product/sigma/l7543<br>rabbit polyclonalanti-Beclin1 (1:1000, #3495, Cell Signaling), https://www.cellsignal.com/products/primary-antibodies/beclin-1-d40c5-rabbit-mab/3495?site-search-type=Products&N=4294956287&Ntt=3495%2C&fromPage=plp&_requestid=2730050<br>rabbit polyclonalanti-Atg5 (1:1000, #12994, Cell Signaling), https://www.cellsignal.com/products/primary-antibodies/atg5-d5f5u-rabbit-mab/12994<br>rabbit polyclonal anti-FOXO3a (1:1000, #2497, Cell Signaling Technology), https://www.cellsignal.com/products/primary-antibodies/foxo3a-75d8-rabbit-mab/2497<br>rabbit polyclonal anti-total-Smad2/3 (1:1000, #13820, Cell Signaling), https://www.cellsignal.com/products/primary-antibodies/phospho-smad1-ser463-465-smad5-ser463-465-smad9-ser465-467-d5b10-rabbit-mab/13820<br>rabbit polyclonal anti-phospho-Smad2/3 (1:1000, #8828, Cell Signaling), https://www.cellsignal.com/products/primary-antibodies/phospho-smad2-ser465-467-smad3-ser423-425-d27f4-rabbit-mab/8828<br>rabbit polyclonal anti-phospho-S6K1 (1:1000, #9205, Cell Signaling), https://www.cellsignal.com/products/primary-antibodies/phospho-p70-s6-kinase-thr389-antibody/9205<br>rabbit polyclonal anti-Deptor (1:1000, Cat# ABS222, Sigma-Aldrich), https://www.sigmaaldrich.com/FR/fr/product/mm/abs222<br>rabbit polyclonal anti-phospho-4E-BP1 (1:1000, #2855, Cell Signaling) https://www.cellsignal.com/products/primary-antibodies/phospho-4e-bp1-thr37-46-236b4-rabbit-mab/2855<br>mouse monoclonalanti-actin (1:6000, A5441, Sigma), https://www.sigmaaldrich.com/FR/fr/product/sigma/a5441<br>mouse monoclonal anti-LacI antibody (05-503I, Sigma-Aldrich), https://www.sigmaaldrich.com/FR/fr/product/mm/05503i

Animals and other research organisms

Policy information about studies involving animals; ARRIVE guidelines recommended for reporting animal research, and Sex and Gender in Research

Laboratory animals	Young (2-3-month-old) and aged (18-22-month-old) C57BL/6JRj male mice were obtained from Janvier Labs (France). Eight-week old C57BL6NTac male mice were obtained from Taconic Biosciences only for depression experiments.Housing conditions were as follows: dark/light cycle from 7am to 9pm, controlled temperature 20-24C, humidity 60% minimum to 70% maximum.
Wild animals	This study did not involve wild animals.
Reporting on sex	Findings involving in vivo experiments apply only to male mice.
Field-collected samples	The study did not involve samples collected from the field.
Ethics oversight	All animal procedures were performed in accordance with French legislation and in compliance with the European Communities Council Directives (2010/63/UE), according to the regulations of Institut Pasteur Animal Care Committees.

Note that full information on the approval of the study protocol must also be provided in the manuscript.

Clinical data

Policy information about clinical studies
All manuscripts should comply with the ICMJE guidelines for publication of clinical research and a completed CONSORT checklist must be included with all submissions.

Clinical trial registration	*Provide the trial registration number from ClinicalTrials.gov or an equivalent agency.*
Study protocol	*Note where the full trial protocol can be accessed OR if not available, explain why.*
Data collection	*Describe the settings and locales of data collection, noting the time periods of recruitment and data collection.*
Outcomes	*Describe how you pre-defined primary and secondary outcome measures and how you assessed these measures.*

This checklist template is licensed under a Creative Commons Attribution 4.0 International License, which permits use, sharing, adaptation, distribution and reproduction in any medium or format, as long as you give appropriate credit to the original author(s) and the source, provide a link to the Creative Commons license, and indicate if changes were made. The images or other third party material in this article are included in the article's Creative Commons license, unless indicated otherwise in a credit line to the material. If material is not included in the article's Creative Commons license and your intended use is not permitted by statutory regulation or exceeds the permitted use, you will need to obtain permission directly from the copyright holder. To view a copy of this license, visit http://creativecommons.org/licenses/by/4.0/
