# Supplementary material for: Systemic GDF11 attenuates depression-like phenotype in aged mice via stimulation of neuronal autophagy
Source: Nat Aging. 2023 Feb 2;3(2):213–28. doi: 10.1038/s43587-022-00352-3 (PMC10154197; doi:10.1038/s43587-022-00352-3)

FoxO3a/Actin

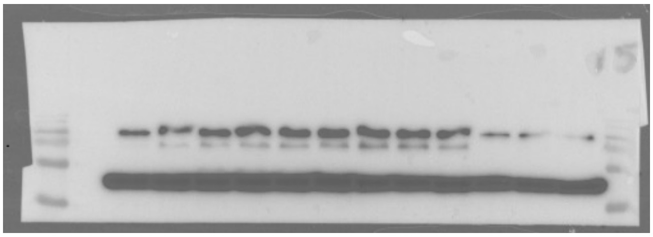

FoxO3a/**Actin**

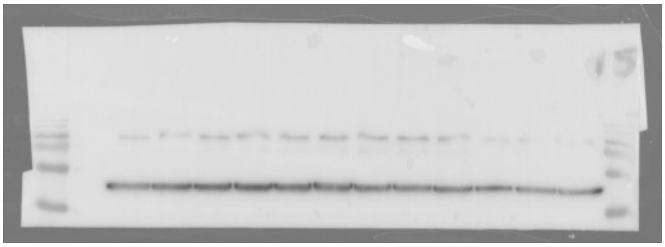

Beclin 1

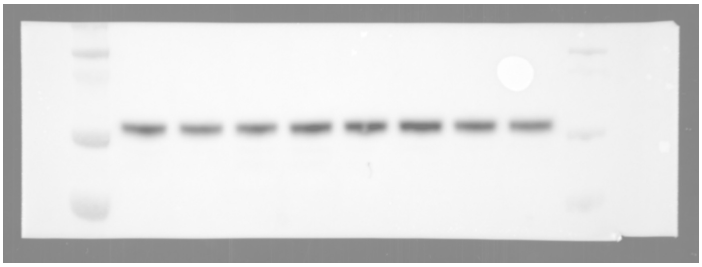

Actin

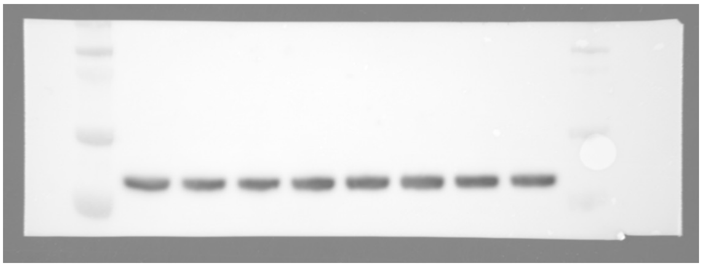

LC3 (up)/**Actin**(down) 1sec

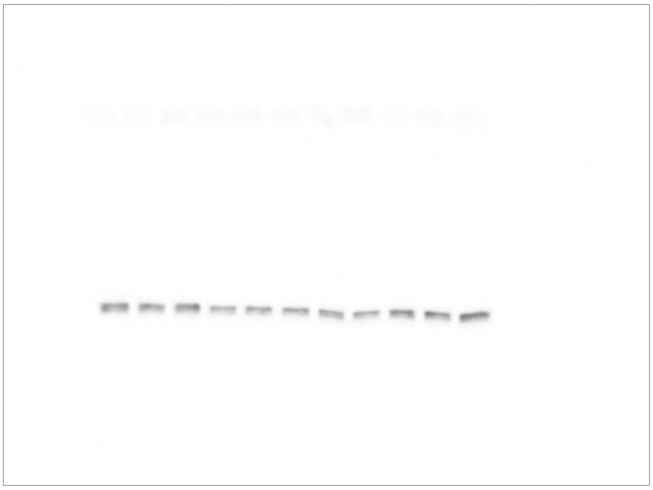

LC3 (up)/Actin(down) 45sec

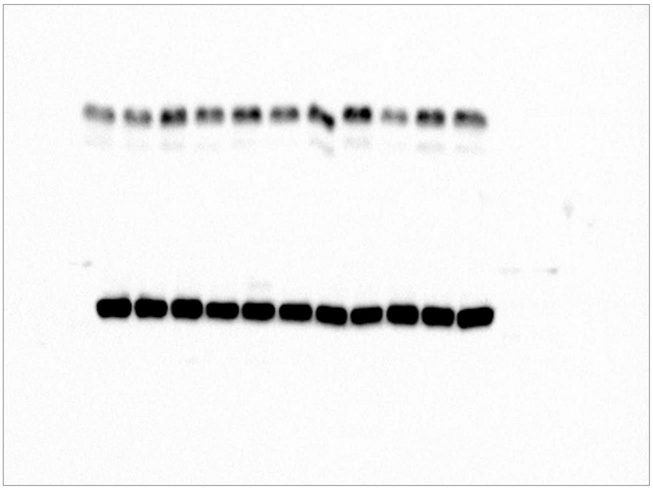

LC3 (up)/Actin(down) long exposure

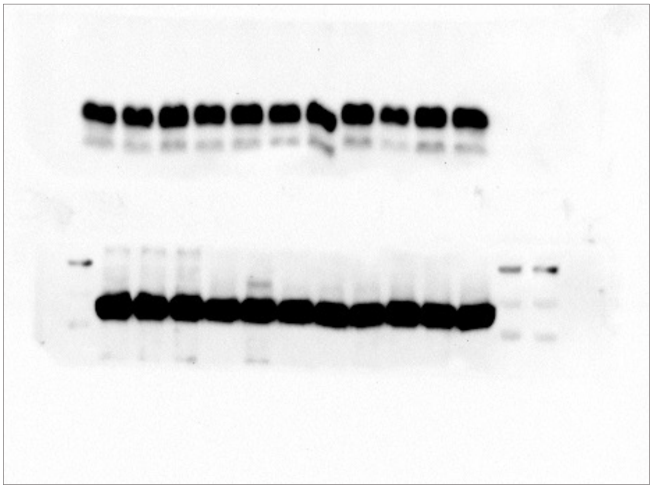

Supplement: Source Data Fig. 2 — Unprocessed western blots [file 43587_2022_352_MOESM4_ESM.pdf]
