# Supplementary figures and images for: Systemic GDF11 attenuates depression-like phenotype in aged mice via stimulation of neuronal autophagy
Source: Nat Aging. 2023 Feb 2;3(2):213–28. doi: 10.1038/s43587-022-00352-3 (PMC10154197; doi:10.1038/s43587-022-00352-3)

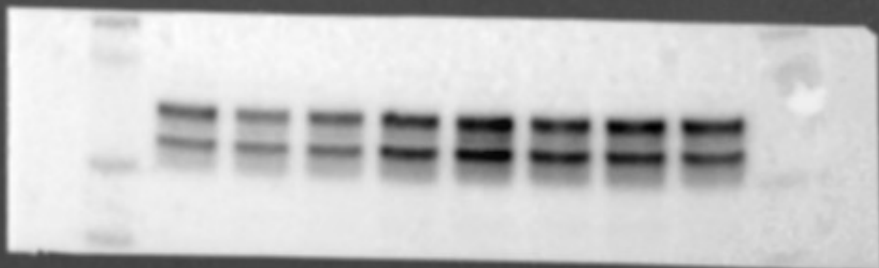

**pSMAD2/3**

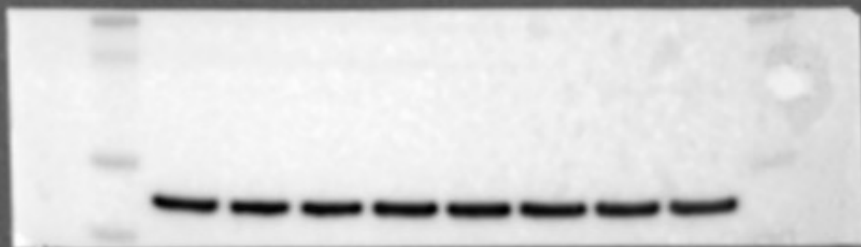

**Actin**

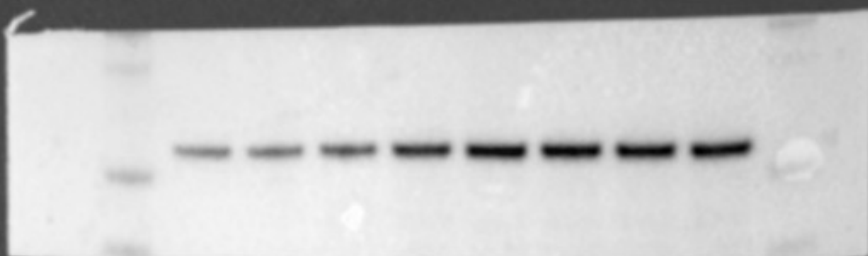

**Beclin 1**

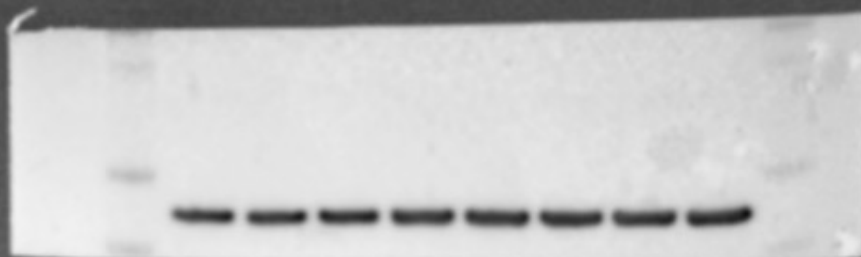

**Actin**

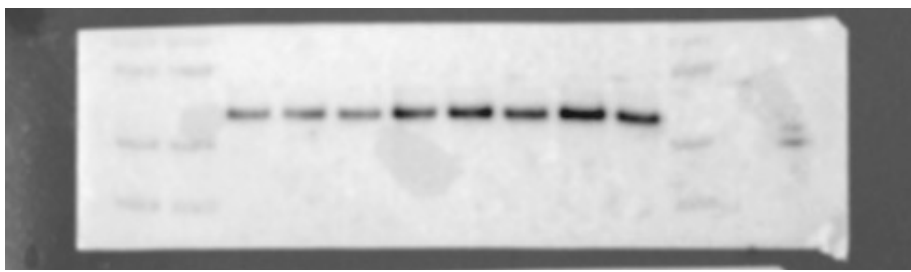

**p62**

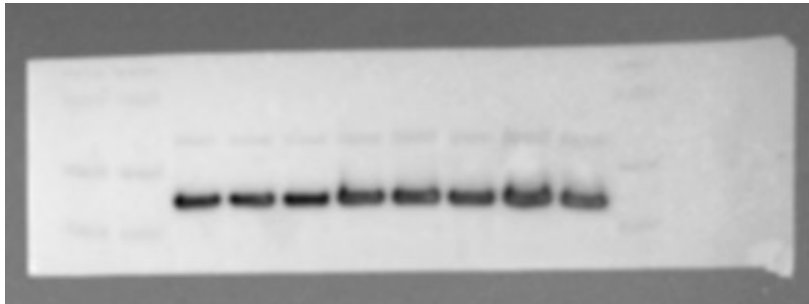

**Actin**

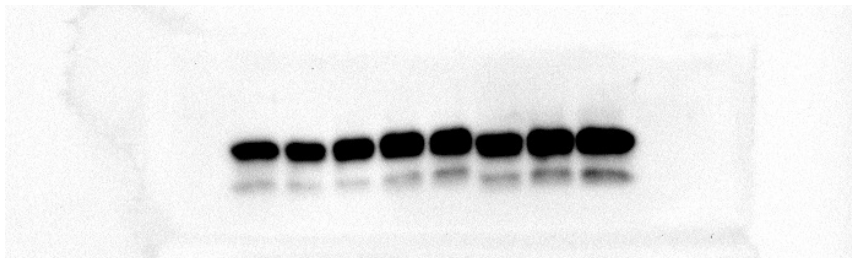

**LC3 long exposure**

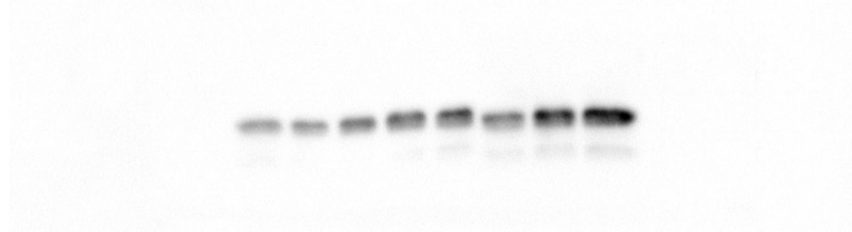

**LC3 short exposure**

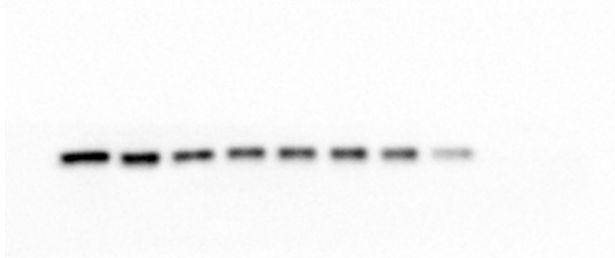

**Actin**

Supplement: Source Data Fig. 3 — Unprocessed western blots [file 43587_2022_352_MOESM6_ESM.pdf]

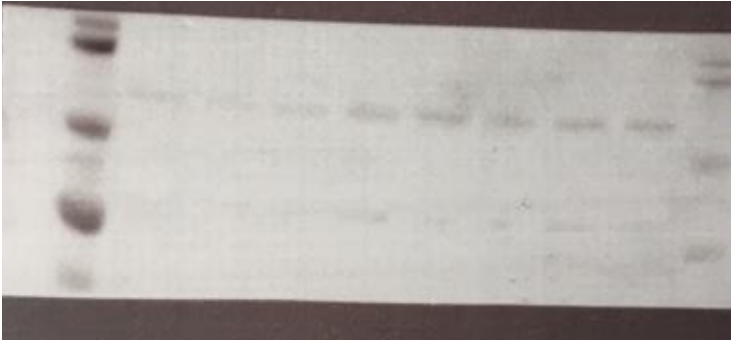

**Deptor**

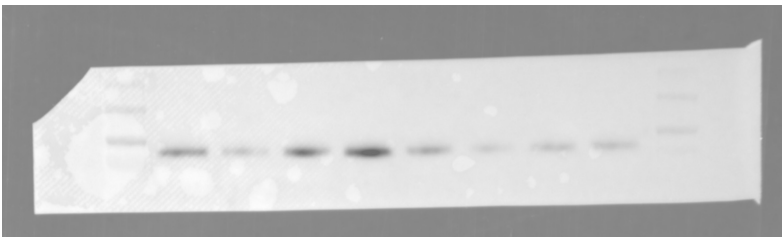

**pS6K1**

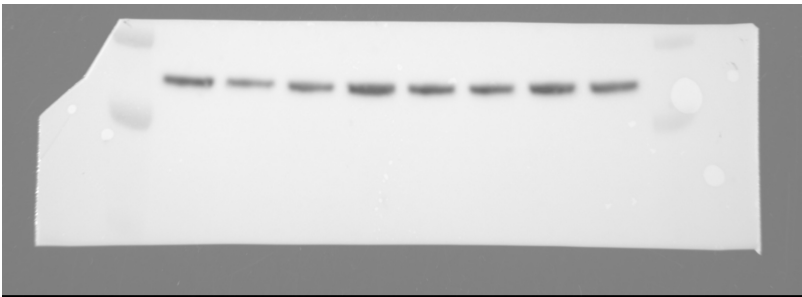

**Actin**

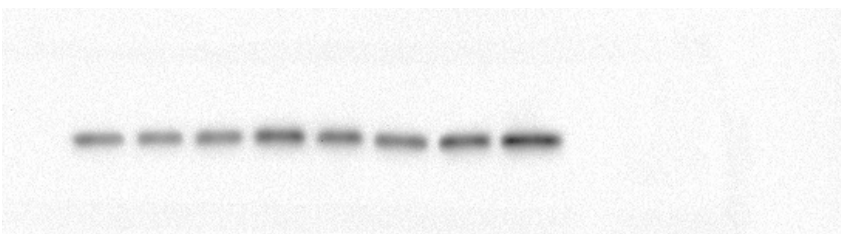

**4E-BP1**

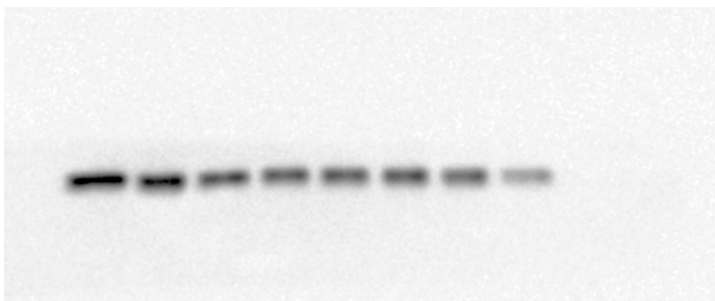

**Actin**

Supplement: Source Data Fig. 4 — Unprocessed Western blots [file 43587_2022_352_MOESM8_ESM.pdf]

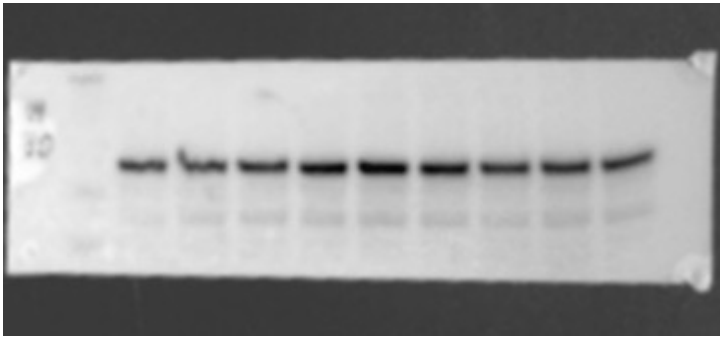

**Beclin 1**

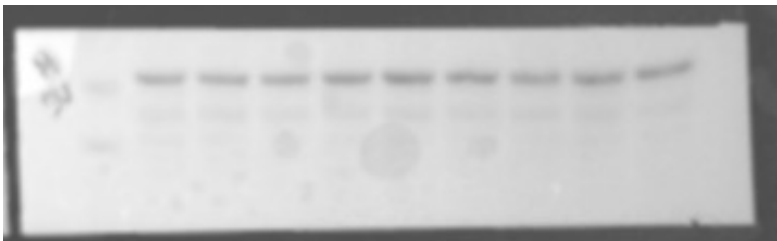

**Atg5**

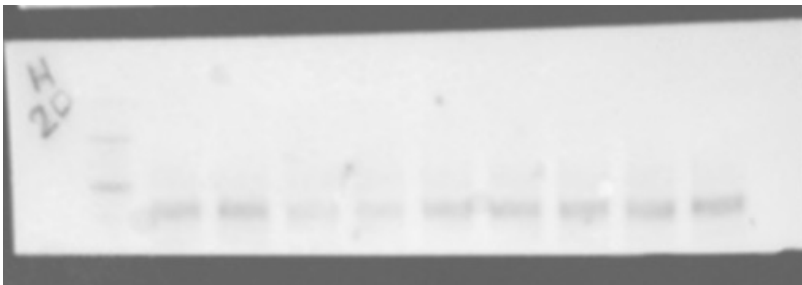

**Lamp1**

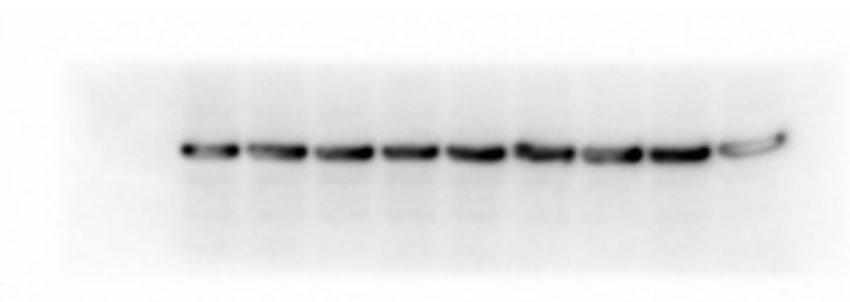

**Actin**

Supplement: Source Data Extended Data Fig. 3 — Unprocessed western blots [file 43587_2022_352_MOESM13_ESM.pdf]
